# The Inflammatory Transcription Factors NFκB, STAT1 and STAT3 Drive Age-Associated Transcriptional Changes in the Human Kidney

**DOI:** 10.1371/journal.pgen.1005734

**Published:** 2015-12-17

**Authors:** Zach K. O’Brown, Eric L. Van Nostrand, John P. Higgins, Stuart K. Kim

**Affiliations:** 1 Department of Developmental Biology, Stanford University, Stanford, California, United States of America; 2 Department of Genetics, Stanford University, Stanford, California, United States of America; 3 Cancer Biology Program, Stanford University, Stanford, California, United States of America; 4 Department of Cellular and Molecular Medicine, University of California, San Diego, La Jolla, California, United States of America; 5 Department of Pathology, Stanford University Medical Center, Stanford, California, United States of America; The University of North Carolina at Chapel Hill, UNITED STATES

## Abstract

Human kidney function declines with age, accompanied by stereotyped changes in gene expression and histopathology, but the mechanisms underlying these changes are largely unknown. To identify potential regulators of kidney aging, we compared age-associated transcriptional changes in the human kidney with genome-wide maps of transcription factor occupancy from ChIP-seq datasets in human cells. The strongest candidates were the inflammation-associated transcription factors NFκB, STAT1 and STAT3, the activities of which increase with age in epithelial compartments of the renal cortex. Stimulation of renal tubular epithelial cells with the inflammatory cytokines IL-6 (a STAT3 activator), IFNγ (a STAT1 activator), or TNFα (an NFκB activator) recapitulated age-associated gene expression changes. We show that common DNA variants in *RELA* and *NFKB1*, the two genes encoding subunits of the NFκB transcription factor, associate with kidney function and chronic kidney disease in gene association studies, providing the first evidence that genetic variation in NFκB contributes to renal aging phenotypes. Our results suggest that NFκB, STAT1 and STAT3 underlie transcriptional changes and chronic inflammation in the aging human kidney.

## Author Summary

The structure and function of human kidneys deteriorate steadily with age, yet little is known about the underlying causes of kidney aging. In this work, we first used a genomics approach to identify candidate regulators of gene expression changes in the aging human kidney and identified inflammation-related transcription factors NFκB, STAT1 and STAT3 as the top candidate regulators. We found that kidney aging is associated with activation of NFκB, STAT1 and STAT3 in the renal parenchyma, and that the gene expression signatures evoked by activation of these transcription factors in human renal epithelial cells mimics age-associated gene expression changes in the kidney. Furthermore, we identified specific genetic variants in the NFκB transcription factor genes *RELA* and *NFKB1* that associate with renal function and chronic kidney disease in humans, implicating NFκB as a potential contributor to the pathogenesis of chronic kidney disease and renal dysfunction in old age. Our findings suggest that activation of the inflammatory transcription factors STAT1, STAT3 and NFκB underlie transcriptional changes and reduced renal function in the elderly.

## Introduction

The human kidney shows a steady and quantifiable decline in function with age, accompanied by stereotyped changes in gene expression and histopathology [1]. The glomerular filtration rate (GFR), a clinical measure of kidney function, shows a steady decline in most individuals starting at about age 40 [2,3]. Kidney aging is an important public health problem because the age-related decline in GFR can lead to chronic kidney disease and progression to end-stage renal disease [3,4]. Chronic kidney disease is a common age-related condition; 35–40% of people over the age of 70 in the United States have some form of chronic kidney disease [3]. Moreover, reduced kidney function is associated with an increased risk of cardiovascular disease [5].

Kidney aging is accompanied by several characteristic changes in renal histopathology including tubulointerstitial fibrosis (extracellular matrix accumulation in the tubulointerstitial space), tubular atrophy, hyaline arteriosclerosis (thickening/hardening of renal arteriole walls), and glomerulosclerosis (scarring of glomerular capillaries and mesangial expansion)[2,6]. Moreover, the cell senescence marker p16/INK4A increases with age in the human kidney, and correlates with interstitial fibrosis and glomerulosclerosis among older individuals [7]. These age-related changes in histopathology and cell senescence might underlie a decrease in kidney function with aging. However, the underlying molecular mechanisms that lead to age-associated changes in renal function and histopathology remain poorly characterized.

The process of kidney aging has begun to be defined at a molecular level by identifying the global transcriptional changes that occur during aging [1,8]. In one study, DNA microarrays were used to profile gene expression in 74 kidneys aged 27 to 92, and identified hundreds of genes that significantly change expression with age [1]. Notably, the gene expression signature for aging was correlated not only with the chronological age of the subjects, but also with the physiologic age of their kidneys, as determined by a histological score called the chronicity index. The chronicity index is an aggregate score of three histopathological measurements of renal aging in sections of renal tissue: renal interstitial fibrosis, tubular atrophy/hyaline arteriosclerosis and glomerulosclerosis. Importantly, the expression of kidney age-related genes correlated with the chronicity index adjusted for chronological age, such that an individual with a low chronicity index tended to have a renal gene expression profile characteristic of a younger individual, whereas an individual with a higher chronicity index tended to have a renal gene expression profiles characteristic of an older individual [1]. Thus, gene expression changes in the aging kidney not only reflect chronological age, but they correlate well with age-related changes in renal histopathology. These studies helped define how changes in gene expression may underlie deterioration of the structure and decline in function of the kidney in old age, but the upstream drivers of these changes in gene expression were previously unknown. With the availability of large genomic datasets from the ENCODE consortia, it is now possible to interrogate transcription factor ChIP-seq binding datasets to systematically identify candidate regulators of gene expression profiles [9–12].

In this study, we took an unbiased genomics approach to search for regulators of age-associated gene expression changes in the kidney, and identified the inflammation-related transcription factors STAT1, STAT3, and NFκB as top candidates. These three transcription factors become activated in old age and can account for a large fraction of the gene expression changes that occur during kidney aging. DNA polymorphisms in the two genes that encode subunits of the canonical NFκB transcription factor (*RELA* and *NFKB1*) are associated with variation in kidney function and chronic kidney disease risk, providing genetic evidence that increasing NFκB activity in old age may play a causal role in age-related renal functional decline. This study highlights the dominant role played by STAT1, STAT3 and NFκB in mediating transcriptional changes in the aging human kidney. Because these transcription factors mediate inflammatory responses, our results suggest that chronic inflammation underlies structural and functional decline of the kidney in old age.

## Results

Previous work identified 630 genes that significantly change expression with age in the cortex and medulla of the kidney during kidney aging [1]. The expression levels of these genes were informative not only for chronological age, but also the physiological state of the kidney as measured by a histopathological score called the chronicity index. The physiologic age of a kidney can also be measured by GFR, a measure of renal function that decreases with age in most people. Therefore, genes related to differences in GFR might be particularly important for physiologic aging of the kidney. In order to evaluate whether the age-related genes are informative for GFR independent of chronological age, we re-analyzed data from [1]. We selected 439 genes that had previously been shown to significantly change with age in the renal cortex, the region of the kidney that contains glomeruli. For these 439 age-related genes, we re-analyzed their expression using a linear regression model containing both age and GFR as covariates. For age-related genes that are informative for renal function, the magnitude of the age coefficient will shrink when GFR is added as a covariate in the regression model. For age-related genes that are not informative for renal function, the age-coefficient should change little between the two regression models. We found that the magnitude of the age coefficient was reduced for 74% of the age-related genes when glomerular filtration rate was included in the regression model (S1 Fig), indicating that many of the age-related genes are informative for renal function.

### NFκB, STAT1 and STAT3 bind kidney age-related genes

**Fig 1:**
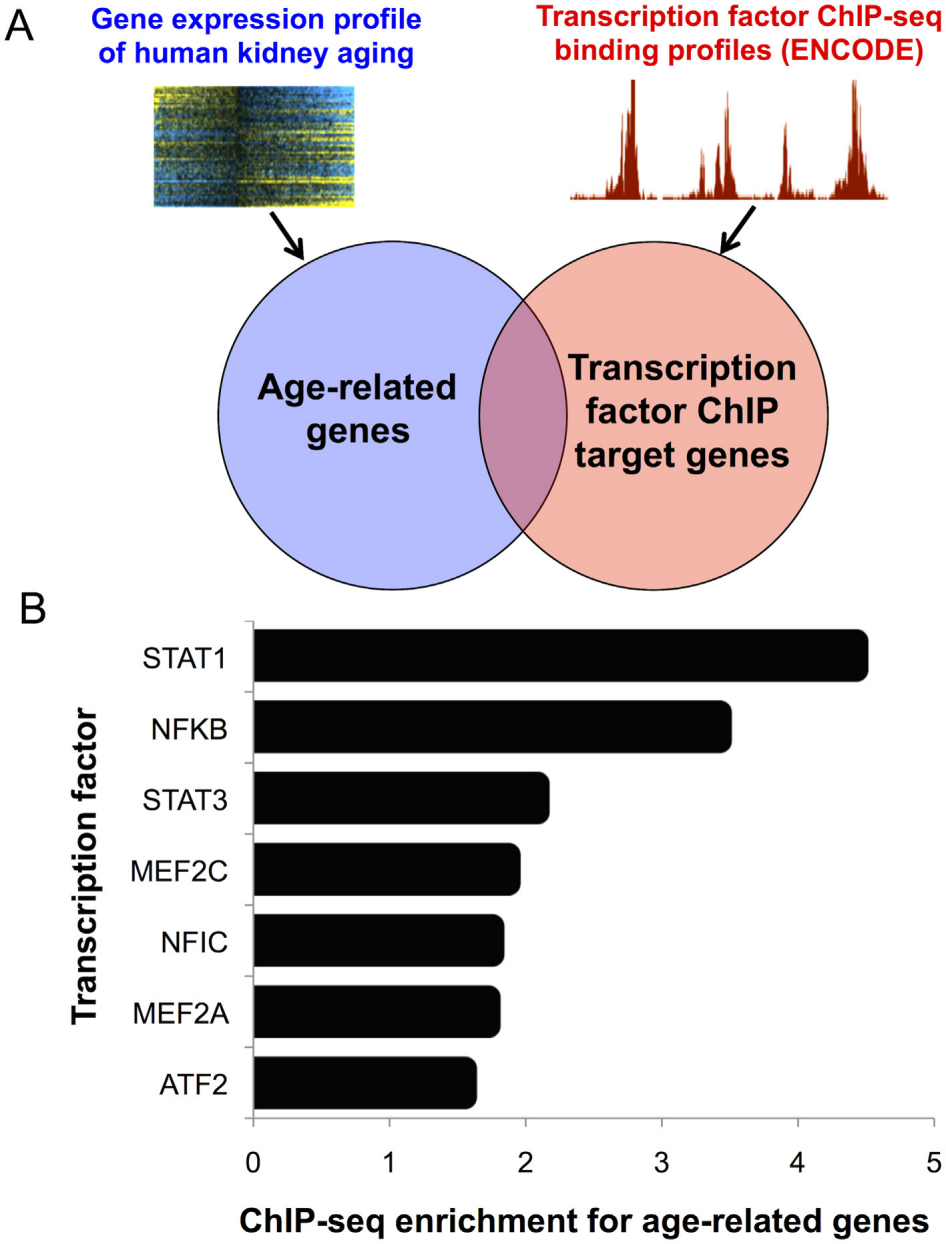
Genomic search for candidate regulators of the kidney aging transcriptome. A. Schematic of the genomic pipeline used to identify associations between transcription factor ChIP-seq binding targets (ENCODE) and kidney age-related genes. The list of age-related genes was overlapped with the ChIP-seq binding targets of 161 transcription factors from 961 ChIP-seq experiments (see Methods). The overlap between the target genes assigned to each transcription factor with age-related genes was determined for each ChIP-seq dataset. B. The histogram shows the fold-enrichments of seven transcription factors that were >1.5 fold enriched and statistically significant after Bonferroni correction (p < 5 x 10^−5^) for binding the kidney age-related genes (see Methods). The three inflammation-associated transcription factors STAT1, NFκB (RelA) and STAT3 showed the strongest ChIP-seq enrichments for binding kidney age-related genes.

To identify mechanisms underlying human kidney aging, we searched for transcription factors that bind nearby or within genes whose expression levels change with age in the kidney. In this study, we examined data for 961 ChIP-seq experiments in diverse human cell lines for 161 transcription factors to screen for datasets in which the set of genes bound by the transcription factor showed a significant overlap with the list of kidney age-related genes, an approach used previously for identifying regulators of aging in *C*. *elegans* (see Methods)(Fig 1A)[10]. Transcription factors in which the magnitude of overlap was >1.5-fold more than expected by chance and statistically significant after Bonferroni correction (p < 5 x 10^−5^) were considered potential regulators of kidney aging, resulting in seven candidate regulators of the kidney aging transcriptome (Fig 1B). The three transcription factors with the strongest enrichments for binding kidney age-related genes from ChIP-seq datasets were STAT1 (4.4-fold-enriched, p = 1.3 x 10^−5^), STAT3 (2.1-fold enriched, p = 3 x 10^−10^) and NFκB (3.4-fold-enriched, p = 7.6 x 10^−17^). STAT1, STAT3 and NFκB were consistently enriched for binding kidney age-related genes in ChIP-seq experiments from different human cell lines (S1 Table).

To examine the specificity of STAT1, STAT3 and NFκB for kidney aging, we asked whether these three transcription factors would show a strong enrichment for binding to diverse sets of genes from microarray expression data in the Gene Expression Omnibus (GEO). We selected gene expression datasets from GEO to generate lists of significantly differentially-regulated genes from each expression dataset (p < 0.001). Two of the datasets were from microarray studies of human renal diseases (diabetic kidney disease and nephrosclerosis) and 18 datasets were selected at random from GEO microarray datasets for human Affymetrix U133 DNA chips. For each set of differentially-regulated genes, we determined whether the ChIP-seq binding peaks for STAT1, STAT3 and NFκB showed a significant overlap (defined as >1.5-fold enrichment, p < 5 x 10^−5^ and in the top 5% of most significantly-enriched ChIP-seq datasets). We found only two instances in which the ChIP-seq binding targets of STAT1, STAT3 or NFκB showed a significant overlap; specifically, NFκB ChIP-seq targets from lymphoblastoid cell lines showed a strong overlap with genes that change expression in diabetic kidney disease and STAT3 ChIP-seq targets from MCF-10 breast cancer cell lines showed an overlap with genes that are differentially regulated upon knockdown of PGC-1α in melanoma cell lines (S2 Table). The association between NFκB and diabetic kidney disease is consistent with previous work demonstrating that NFκB and its target genes are activated in human diabetic kidney disease [13]. These results indicate that STAT1, STAT3 and NFκB do not generally show a significant enrichment for binding differentially-regulated genes from most microarray expression datasets.

### A common transcriptional profile of kidney aging in humans and rats

We compared the transcriptional profile of kidney aging from humans to a kidney aging transcriptional profile from rats [14]. We identified genes from the rat that were both orthologous to the 630 human kidney age-related genes and showed expression in the rat aging microarray experiment, resulting in a list of 427 genes. Next, we compared the age-related changes in expression of these 427 genes in human kidneys with the age-related changes in expression of the orthologous genes in rat kidneys, and found a significant correlation between their age-related changes in human and rat kidneys (r = 0.46, p < 10^−5^)(S2 Fig). 114 of these 427 genes were significantly age-related in both human and rat kidneys (6.7-fold enrichment, p < 10^−40^)(S3 Table). This result indicates that there is a common transcriptional signature of kidney aging in humans and rats. Since the human kidney samples were mainly obtained from diseased patients (mostly individuals with renal cell carcinomas), whereas the rat samples were obtained from a normal aging cohort, the similarity between the human and rat kidney aging transcriptome indicates that the human gene expression pattern captures true age-related changes.

Next, we asked whether STAT1, STAT3 and NFκB bound to the common set of human and rat kidney aging genes; specifically, we calculated the overlap between the ChIP-seq binding peaks in the ENCODE datasets for these three transcription factors with the list of 114 kidney aging genes shared between human and rat. The ChIP-seq binding targets for STAT1, STAT3 and NFκB were significantly enriched for binding these 114 kidney age-related genes, suggesting that these inflammatory transcription factors may contribute to age-related gene expression changes in both humans and rats (S4 Table).

### Activity of STAT1, STAT3 and NFκB increases with age in the kidney

STAT1 and STAT3 belong to the highly conserved JAK/STAT family of transcription factors. The activation and nuclear localization of STAT transcription factors is primarily regulated by tyrosine phosphorylation, dimerization and translocation to the nucleus in response to specific cytokines or growth factors [15,16]. Inducers of the STAT transcription factors include inflammatory cytokines, such as interleukin-6 (IL-6) (primarily an activator of STAT3) or interferon gamma (IFNγ) (primarily an activator of STAT1) [16,17]. Similarly, the canonical NFκB transcription factor, a heterodimer of p50 (encoded by the *NFKB1* gene) and p65/RelA (encoded by the *RELA* gene) can be activated by inflammatory cytokines, such as tumor necrosis factor alpha (TNFα) and interleukin-1 beta (IL-1ß), as well as DNA damage, lipopolysaccharide, or reactive oxygen species [18,19].

**Fig 2:**
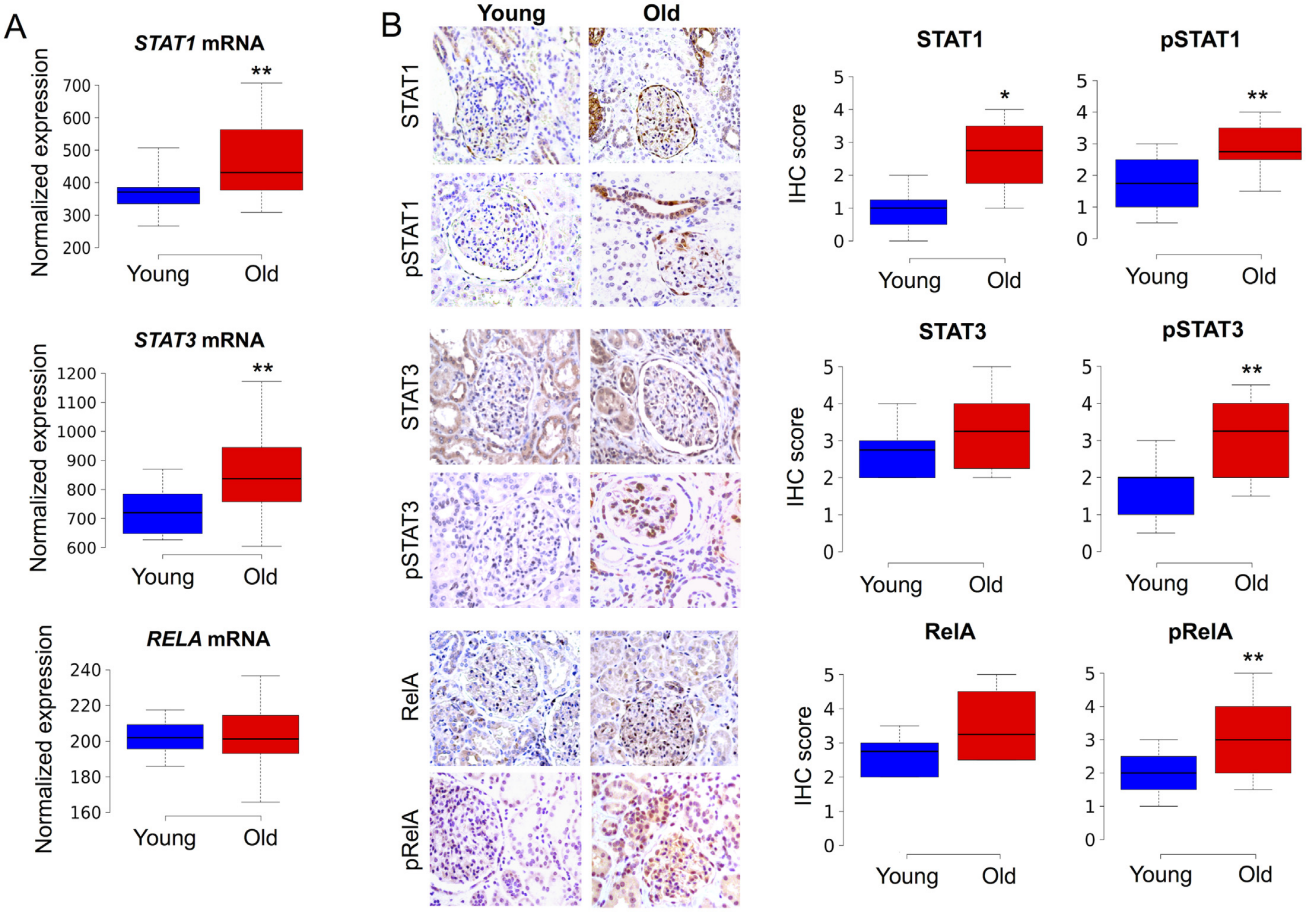
Increased activity of STAT1, STAT3 and NFκB transcription factors during kidney aging. A. Boxplots showing significantly increased mRNA expression of *STAT1* and *STAT3* in old (> 65 years, n = 42) versus young (< 45 years, n = 10) renal cortex samples. There was not a significant difference in *RELA* mRNA expression levels between old and young individuals. The normalized gene expression data were from a previously published microarray study [1]. B. Left panels: Images of IHC showing immunoreactivity for STAT1, pSTAT1, STAT3, pSTAT3, RelA and pRelA in sections of renal cortex from young (<40 years) and old (>65 years) individuals. Positive signal is represented by brown color. Right panels: Boxplots showing IHC immunoreactivity scores for STAT1 (n = 10 young, n = 9 old), nuclear pSTAT1 (n = 7 young, n = 10 old), STAT3 (n = 6 young, old n = 8), nuclear pSTAT3 (n = 10 young, n = 10 old), RelA (n = 6 young, n = 6 old) and nuclear pRelA (n = 10 young, n = 10 old). The boxes indicate boundaries for the 25th and 75th percentile, and lines indicate maximum, median and minimum values. ** Indicates p < 0.01, * p < 0.05 (one-sided Mann-Whitney U test).

To test if the three candidate regulators (STAT1, STAT3 and NFκB) are responsible for driving gene expression changes during kidney aging, we assessed the expression levels and activity of these transcription factors in young and old human kidney tissues. We first examined the age-associated changes in the mRNA expression levels of the genes encoding these three transcription factors, using previously published microarray gene expression data [1]. We found a significant age-related increase in the mRNA expression levels of *STAT1* (1.23-fold increase, p = 0.006, Student’s t-test) and *STAT3* (1.2-fold increase, p = 0.008, Student’s t-test) in old versus young kidneys, but no significant change in *RELA* mRNA expression levels with age (Fig 2A).

We next performed immunohistochemistry (IHC) experiments to assess the localization patterns, expression levels and activities of STAT1, STAT3 and NFκB (RelA) protein in paraffin sections of renal cortex tissues from young (25–44 years of age) and old (66–85 years of age) individuals. STAT1, STAT3 and RelA were predominantly localized to the cytoplasm with sporadic nuclear staining in a small fraction of tubular epithelial and glomerular cells. Expression of these three transcription factors was observed in multiple renal cell types, including tubular epithelial, glomerular and interstitial cells (Fig 2B). We found a significant increase in overall STAT1 immunoreactivity with age (p = 0.02, Mann-Whitney U test), but no significant change in STAT3 or RelA immunoreactivity with age.

To assess differences in transcription factor activity between old and young kidneys, we used antibodies that recognize activated (phosphorylated) forms of these transcription factors (herein abbreviated as pSTAT1, pSTAT3 and pRelA). We observed significantly higher nuclear levels of pSTAT1 (p = 0.002, Mann-Whitney U test), pSTAT3 (p = 0.003, Mann-Whitney U test) and pRelA (p = 0.006, Mann-Whitney U test) in old versus young renal cortex (Fig 2B). Activation of these three transcription factors was observed in multiple epithelial cell types in aged kidneys. Immunoreactivity for pSTAT1 was restricted to subsets of tubules, a few cells within glomeruli, and Bowman’s capsule epithelial cells. The tissue expression pattern for pSTAT3 in older renal cortex tissues was somewhat broader than that of pSTAT1, and included a larger subset of tubular epithelial cells, glomerular cells and interstitial cells. Expression of pRelA in older kidneys spanned most regions of the renal cortex, including the majority of renal tubular epithelial cells, subsets of glomerular cells and interstitial cells.

### Activation of STAT1, STAT3 and NFκB by inflammatory cytokines recapitulates kidney aging at the transcriptional level

To test if STAT1, STAT3 and NFκB activation drive transcriptional changes of their target genes during kidney aging, we first sought to identify the set of target genes directly regulated by these transcription factors in human renal tubular epithelial cells (HK-2 cells). Genes were considered to be direct targets of each transcription factor if they were directly bound to the transcription factor in ChIP-seq datasets [11] and if their expression was responsive to transcription factor activation by inflammatory cytokine stimulation. We used IFNγ to activate STAT1 signaling, IL-6 to activate STAT3 signaling and TNFα to activate NFκB signaling in HK-2 cells. IFNγ predominantly regulates gene expression via STAT1 activation, as knockdown of STAT1 in IFNγ-stimulated cells represses the vast majority of IFNγ-induced genes [15]. Likewise, IL-6 regulates gene expression predominantly through STAT3, as 86% of IL-6-induced transcriptional changes were suppressed by STAT3 inhibition of IL-6 treated HK-2 cells using the STAT3-specific inhibitor drug S3I-201 (p < 10^−5^, binomial test)(S3 Fig). Finally, about 90% of the transcripts that increase expression in response to TNFα in human cells are NFκB-dependent [17]

**Fig 3:**
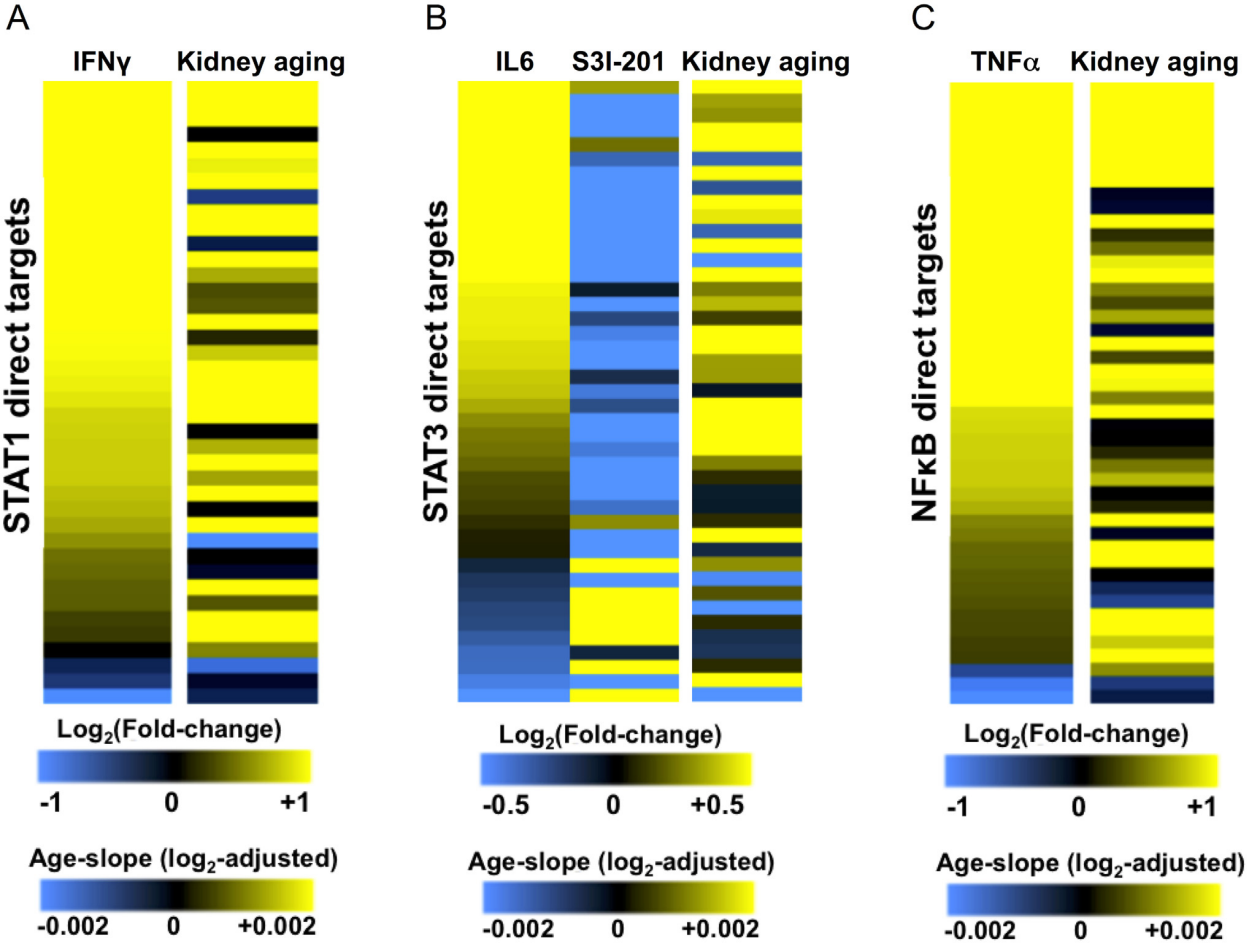
Activation of STAT1, STAT3 or NFκB by inflammatory cytokines recapitulates kidney aging-related gene expression patterns in human renal epithelial cells. A. The left column of the heat map shows the log_2_ fold-changes of 40 direct targets of STAT1 following IFNγ stimulation of HK-2 cells from microarray expression profiling experiments. The right column of the heat map shows the corresponding log_2_-adjusted beta coefficient (age-slope) for these STAT1 direct targets during kidney aging. B. The left column of heat map shows the log_2_ fold-changes of 43 direct targets of STAT3 following IL-6 stimulation of HK-2 cells from microarray expression profiling experiments. The middle column shows changes in expression following treatment with the STAT3 inhibitor S3I-201. The right column shows the corresponding log_2_-adjusted beta coefficient (age-slope) during kidney aging. Aging gene expression data are from [1]. C. The left column of the heat map shows the log_2_ fold-changes of 43 direct targets of NFκB following TNFα stimulation of HK-2 cells from microarray expression profiling experiments. The right column of the heat map shows the corresponding log_2_-adjusted beta coefficient (age-slope) for these NFκB direct targets during kidney aging. Yellow indicates increased gene expression (positive fold-change or age-slope) and blue indicates decreased gene expression (negative-fold change or age-slope).

We used DNA microarrays to profile changes in gene expression following stimulation of HK-2 cells with the inflammatory cytokines IFNγ, IL-6 or TNFα. To define a set of STAT1, STAT3 or NFκB direct target genes we combined transcription factor binding data from ChIP-seq experiments with microarray expression data to identify genes that are bound by the transcription factor and differentially regulated by cytokine stimulation of HK-2 cells (see Methods). We thereby identified 40 STAT1 direct target genes, 43 STAT3 direct targets, and 43 NFκB direct targets (Fig 3; S5 Table).

Next, we compared the expression changes of STAT1, STAT3 and NFκB regulated-targets in HK-2 cells with their expression changes during kidney aging. Expression changes for the direct targets of STAT1 following IFNγ stimulation largely recapitulated their expression changes during aging (Fig 3A). Notably, the majority of STAT1 direct targets that increased expression upon IFNγ stimulation also increased expression during kidney aging (84% concordance, p < 10^−4^, Binomial test). These STAT1-induced direct targets include genes involved in the regulation of apoptosis (e.g. *PML*, *BAK1*, *MX1*), antigen presentation (e.g. *HLA-E*, *TAP1*) and members of the JAK-STAT signaling pathway (e.g. *SOCS3*, *STAT1*, *STAT2*, *STAT3*).

For STAT3, we profiled changes in gene expression upon stimulation of HK-2 cells with IL-6 (for STAT3 activation) or STAT3-specific inhibition by the drug S3I-201 following IL-6 treatment. The expression profile of the 43 STAT3 direct targets following IL-6 stimulation was correlated with the changes in their expression during aging (r = 0.40, p = 0.01) (Fig 3B). The majority of STAT3 targets that were induced by IL-6 were also induced during kidney aging (84% concordance, p < 0.001, Binomial test). To inhibit STAT3 activation, we stimulated HK-2 cells with IL-6 followed by addition of the STAT3 inhibitor S3I-201. Gene expression changes for the STAT3 direct targets following STAT3 inhibition were strongly anti-correlated with their changes in gene expression during kidney aging (r = -0.51, p = 0.003)(Fig 3B). Thus, activation of STAT3 led to a transcriptional response characteristic of kidney aging, while inhibition of STAT3 elicited a transcriptional program characteristic of a younger kidney. The set of 43 STAT3 direct targets include genes known to be involved in the regulation of cell proliferation (e.g. *MYC*), metabolic functions (e.g. *NNMT*, *NAMPT*) and regulation of apoptosis (e.g. *BIRC3*, *BCL6*, *TNFRSF1A*).

Expression changes of the direct target genes of NFκB following TNFα treatment were strongly correlated with their gene expression changes in the aging kidney (r = 0.54, p < 10^−4^)(Fig 3C). 35 out of the 40 NFκB target genes that were induced by TNFα were induced during kidney aging (88% concordance, p < 10^−5^, binomial test). The set of NFκB-direct targets include mediators of innate immune responses (e.g. *LTB*, *ICAM1)*, apoptosis *(e*.*g*. *BIRC3*, *TRAF3)* and feedback regulators of NFκB signaling (e.g. *NFKBIA*, *NFKBIE*, *NFKBID*).

The direct targets of STAT1, STAT3 and NFκB were largely non-overlapping with each other, suggesting that combinatorial activation of these transcription factors might have additive effects on the aging gene expression profile (S4 Fig). To investigate whether simultaneous activation of STAT1, STAT3 and NFκB might have additive effects in promoting the aging transcriptional phenotype, we used DNA microarrays to profile changes in gene expression following simultaneous stimulation of HK-2 cells with each possible combination of IL-6, IFNγ and TNFα. We then compared the resulting changes in gene expression for the direct targets of STAT1, STAT3 or NFκB in HK-2 cells with the changes in their expression during kidney aging. Generally, we found that stimulation of HK-2 cells with two or more of these inflammatory cytokines recapitulated more of the kidney age-induced transcriptional profile than IL-6, IFNγ, or TNFα alone (S5 Fig). There was a particularly strong correlation in the gene expression profile of these genes following simultaneous treatment with the three inflammatory cytokines and their expression behavior during kidney aging (r = 0.51, p < 10^−5^). Notably, the vast majority of direct targets of STAT1, STAT3 and NFκB that were induced by combined treatment with IFNγ, IL-6, and TNFα were also induced during kidney aging (90% concordance, p < 10^−9^, binomial test).

### General responses to inflammatory cytokines recapitulate kidney aging at the transcriptional level

To go beyond the direct targets of STAT1, STAT3 and NFκB, we wanted to ask whether the general transcriptional responses to the IFNγ, IL-6, or TNFα resembled age-associated gene expression changes in the kidney. These target genes may include both direct and indirect targets of the three transcription factors. For each cytokine, we identified genes that were significantly differentially regulated (p < 0.001) in our DNA microarray experiments upon cytokine stimulation of HK-2 cells (see Methods). This analysis identified 48 IFNγ-regulated genes, 32 IL-6-regulated genes and 230 TNFα-regulated genes (S5 Table).

For these cytokine-regulated genes, the kidney age-related gene expression changes were significantly correlated with their transcriptional changes in response to IFNγ (r = 0.64, p < 10^−5^), IL-6 (r = 0.36, p = 0.02) and TNFα (r = 0.46, p < 10^−10^). IFNγ induced a number of canonical interferon responsive genes (*IRF1*, *GBP1*, *GBP2*), and genes involved in innate immune responses (*IL18BP*, *TNFRSF1A*, *ICAM1*). IL-6 induced genes involved in feedback regulation of STAT3 signaling (e.g. *SOCS3*, *STAT3*), as well as inflammation-related transcription factor genes (e.g. *CEBPD*, *BCL6*). TNFα induced several inflammatory cytokine genes (e.g. *IL6*, *IL32*, *IL23A*, *IL8*), chemokine genes (e.g. *CCL2*, *CXCL2*, *CCL20*), complement factors (e.g. *C3*, *CFI*), and regulators of apoptosis (e.g. *FAS*, *TNFAIP3*, *BIRC3*). These results suggest that IFNγ, IL6 and TNFα may contribute to activation of the inflammatory response in the aging kidney.

As a control, we examined whether the overlap between the kidney aging transcriptome and transcriptional responses to IL-6, IFNγ or TNFα are specific to these cytokines or whether other cytokines tend to show a similar effect. We obtained published gene expression datasets from GEO for the transcriptional response of human cells to ten different cytokines and growth factors [20–30]. For each gene expression dataset, we then identified genes that showed a significant change in expression (p < 0.001) following cytokine or growth factor stimulation. Finally, we calculated the correlations between the gene expression changes following cytokine/growth factor treatment and their expression changes during kidney aging. The IL-6, IFNγ and TNFα transcriptional responses showed the most significant correlations with kidney aging from this list (S6 Fig). Of the ten other cytokines, the transcriptional responses to eight did not significantly correlate with the kidney aging gene expression profile and the transcriptional responses to IL-1ß and VEGFA showed a moderate correlation (S6 Fig). The association between kidney aging and the IL-1ß transcriptional response might be expected given that IL-1ß activates NFκB, similar to TNFα [18]. This analysis indicates that the kidney aging transcriptome shows a strong overlap with the transcriptional responses evoked by IFNγ, IL-6, and TNFα, but not with the transcriptional responses evoked by various other cytokines.

### TNFα leads to a mesenchymal cell fate transition in HK-2 cells

A common histopathologic feature of kidney aging is tubulointerstitial fibrosis, a progressive scarring of the kidney, characterized by accumulation of extracellular matrix in the renal interstitial space, and an increased abundance of myofibroblasts, the primary mesenchymal cell type responsible for increased collagen deposition in fibrotic kidneys [31]. One cellular mechanism of interstitial fibrosis is epithelial to mesenchymal transition (EMT), whereby tubular epithelial cells acquire characteristics of fibroblasts and myofibroblasts in response to injury or inflammatory stimuli [31]. Microarray analysis of differentially-regulated genes following TNFα stimulation of HK-2 cells showed increased expression of several genes characteristically expressed in fibroblasts and myofibroblasts, including the EMT transcription factor *TWIST1* and the mesenchymal marker vimentin (S6 Table). These microarray results suggest that TNFα might initiate a mesenchymal differentiation program in HK-2 cells.

**Fig 4:**
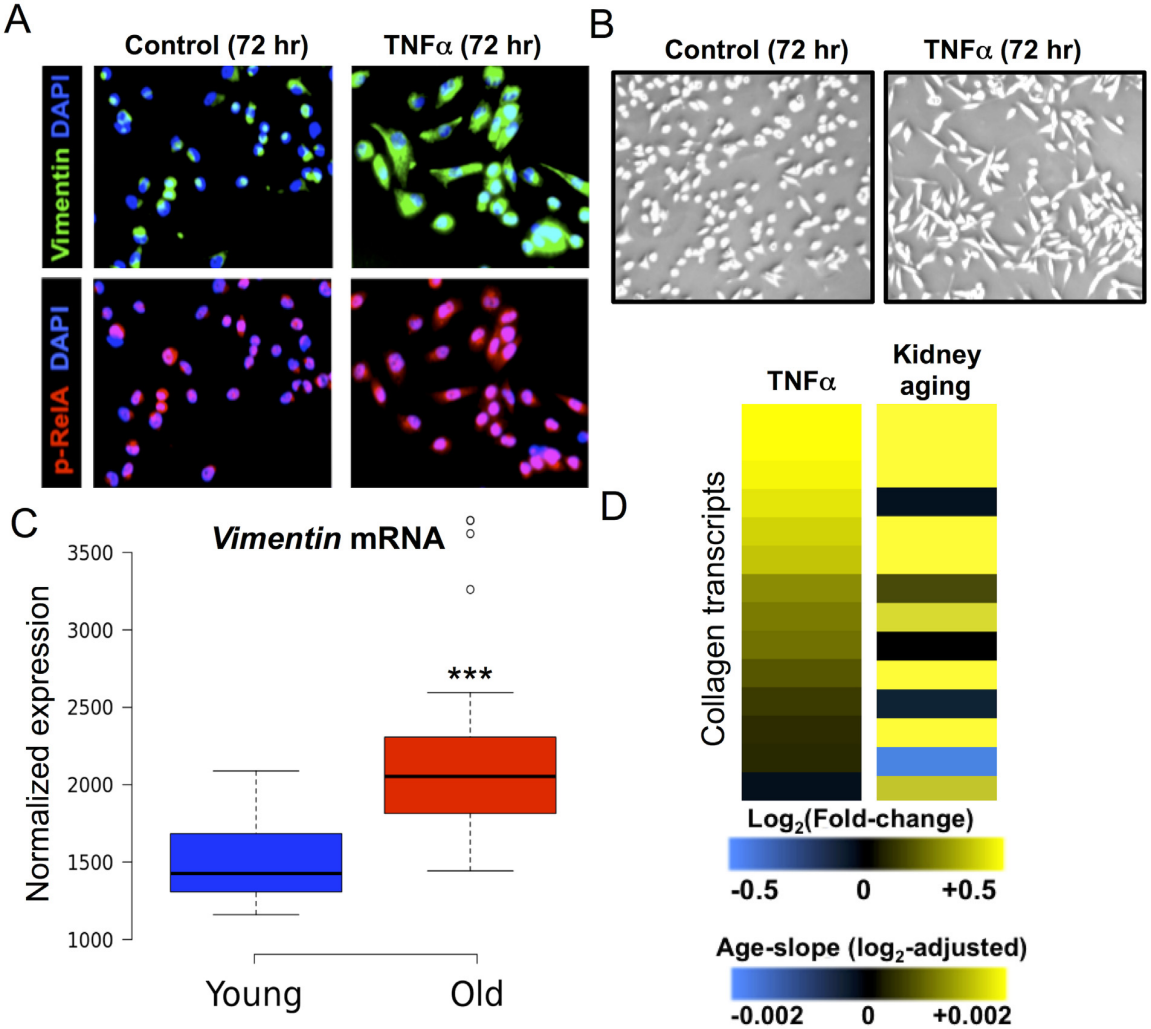
TNFα induces a mesenchymal transition and fibrogenic gene expression in HK-2 cells. A. Immunofluorescence images of HK-2 cells treated with vehicle control (left) or TNFα (right); Cells were co-stained with anti-vimentin antibodies (green, top panels) and pRelA antibodies (red, bottom panels). Increased nuclear pRelA staining in TNFα-treated cells indicates NFκB activation. Induction of vimentin in TNFα-stimulated cells marks the cell fate transition to a mesenchymal-like phenotype. B. Micrograph of HK-2 cells treated with vehicle control (left) or TNFα (right). The TNFα-stimulated cells display an elongated fibroblastic morphology, characteristic of mesenchymal cells. C. The boxplot shows significantly increased levels of vimentin mRNA expression during kidney aging, using expression data from Rodwell et al. 2004. D. Collagen genes are induced by TNFα stimulation and tend to increase expression during aging. The left column of the heat map shows the log_2_-fold-changes in expression of 14 collagen transcripts following stimulation of HK-2 cells with TNFα. The right column of the heat map shows the age-related slope for these genes during kidney aging.

Previous work has shown that NFκB can drive EMT in the context of cancer metastasis. Specifically, TNFα stimulation or overexpression of RelA is sufficient to promote EMT of carcinoma cells [32–34]. We extended these observations by asking whether TNFα could promote a mesenchymal phenotype in HK-2 cells. As expected, TNFα treatment resulted in increased levels of nuclear pRelA, indicating NFκB activation (Fig 4A). We found that prolonged TNFα treatment caused HK-2 cells to acquire an elongated morphology characteristic of mesenchymal cells (Fig 4B), as well as increased protein expression of the mesenchymal marker vimentin (Fig 4A), the expression of which also increases significantly with age (1.43-fold increase, p < 10^−4^)(Fig 4C). These findings indicate that TNFα is sufficient to induce a mesenchymal transition in HK-2 cells, consistent with the results of a previous study [35]. Since renal fibrosis is associated with an increase in extracellular collagen deposition, we asked whether collagen genes tended to be induced by TNFα, and found that the majority of detectable collagen transcripts increased expression following TNFα stimulation and similarly increased during kidney aging (Fig 4D and S6 Table). Taken together, our results suggest that NFκB activation may contribute to the development of interstitial fibrosis in the aging kidney by promoting mesenchymal differentiation and fibrotic gene expression.

### Co-variation in renal STAT1, STAT3 and NFκB activity between individuals

One possibility is that age-associated activation of STAT1, STAT3 and NFκB is caused by a common mechanism (e.g. chronic inflammation), in which case their activities should co-vary in individuals. That is, in individuals of the same age, the levels of STAT1, STAT3 and NFκB transcription factor activity might fluctuate in a coordinated fashion, suggesting that they respond to individual differences in a common upstream mechanism.

In order to ask whether STAT1, STAT3 and NFκB target genes are co-regulated in individual kidney samples, we first devised a measurement to reflect the activity of these transcription factors in different individuals using DNA microarray expression data from 73 kidneys. We used the expression levels of the direct target genes for STAT1, STAT3 and NFκB, respectively, as a proxy for their activity in individual kidney samples. For each renal cortex sample, we calculated a normalized z-score indicating whether expression of the target genes in that individual kidney was higher or lower than the average score for that age.

**Fig 5:**
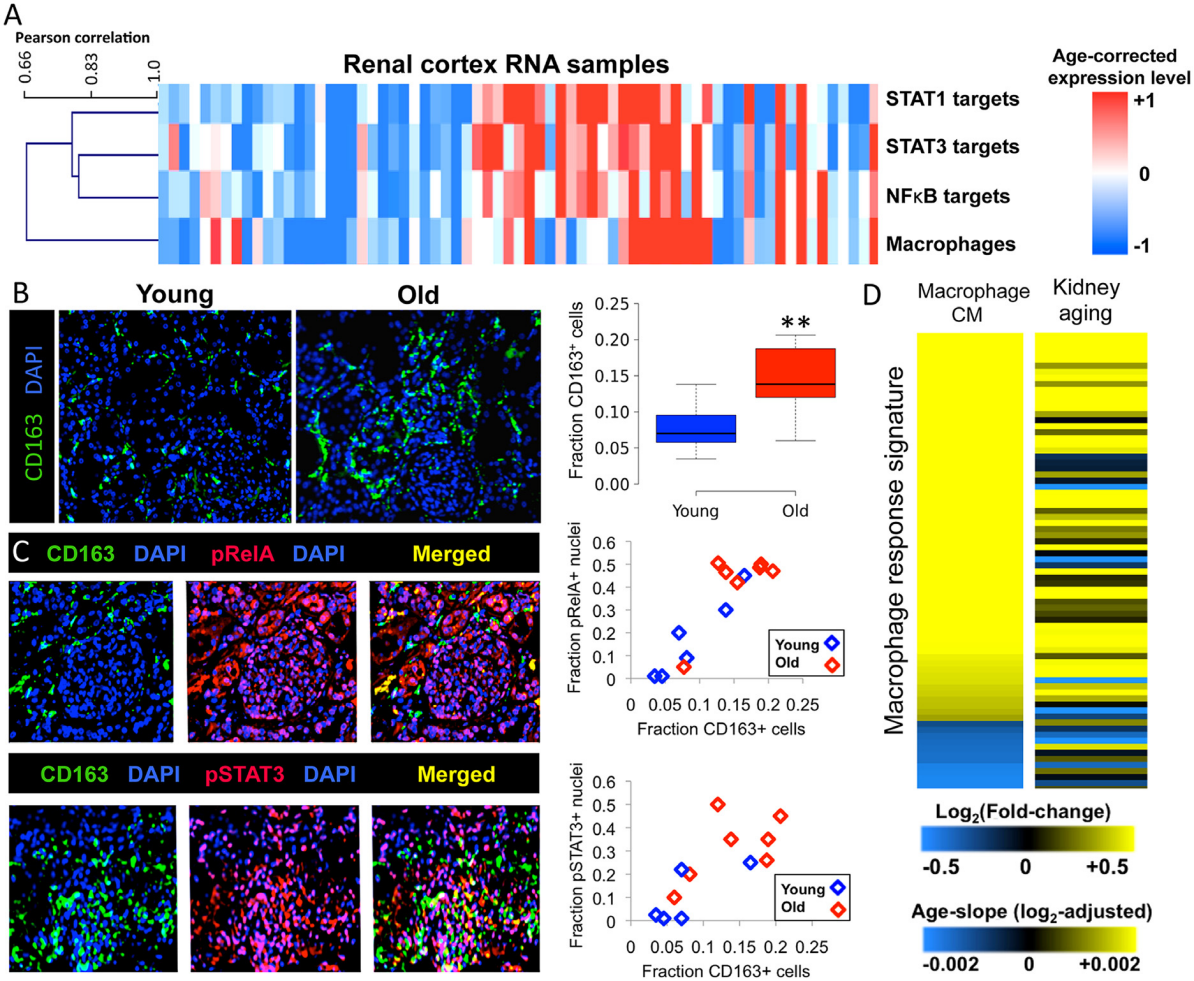
Correlations of the activities of STAT1, STAT3, NFκB and macrophage abundance in individual kidneys. A. In this heat map the rows indicate estimated macrophage abundance based on macrophage-specific transcript expression, or level of activation of each transcription factor, based on the averaged expression of transcription factor direct target genes (see Methods). The colors indicate relative levels of macrophage abundance or transcription factor target gene expression compared to other individuals of that age; red columns represent individuals with higher expression of macrophage-specific transcripts or transcription factor target genes for their age (older than expected at the gene expression level), and blue columns represent individuals with lower expression levels of macrophage markers of transcription factor target genes for their age (more youthful at the gene expression level). The columns are clustered such that individuals with values that are high or low for their age appear together. B. Macrophage infiltration in the kidney increases with age: images of renal cortex samples showing CD163 staining (green) with DAPI counterstaining (blue). Shown is a representative example of a young renal cortex (left) and an old renal cortex (right). The boxplot quantifies macrophage infiltration. The relative abundance of macrophages was defined as the fraction of CD163^+^ cells/all DAPI^+^ cells The boxes indicate 25th and 75th percentiles for the group, and the lines indicate maximum, median and minimum values. **P < 0.01 (Student’s t test, one-sided). C. Macrophage abundance correlates with levels of activation of STAT3 and NFκB in individual kidneys. Human renal cortex stained with anti-CD163 antibodies (macrophage marker), co-stained with either pRelA (top panels) or pSTAT3 antibodies (bottom panels). Right scatterplots show the correlation between overall macrophage abundance and pSTAT3 nuclear immunoreactivity (top) or pRelA nuclear immunoreactivity (bottom) in epithelial compartments of individual renal cortex tissues. Red diamonds represent old individuals (ages 66–85 years) and blue diamonds represent young individuals (ages 25–44 years). The x-axis indicates the fraction of cells that are CD163^+^ and the y-axis indicates for the fraction of cells stained positive for pRelA or pSTAT3. D. Macrophage-conditioned media responsive genes are induced during kidney aging. The left column of the heat map shows 77 differentially regulated genes in response to macrophage-conditioned medium (S7 Table) from two microarray studies in different human cell lines [38,39]. The right column of the heat map shows the changes in gene expression for these genes during kidney aging (age-related slope).

We then compared the activity scores for the three transcription factors to each other, and found that there was a strong correlation in the age-adjusted activity levels of STAT1, STAT3 and NFκB in different individuals (Fig 5A). This result shows that the activities of these three transcription factors co-vary in individuals, such that those with relatively high expression of NFκB target genes also tend to have relatively high expression of STAT1 and STAT3 target genes for their age. These results suggest that there might be a common mechanism underlying the coordinate regulation of these three transcription factors.

### Correlation between transcription factor activity and macrophage infiltration

We hypothesized that macrophages might contribute to coordinated activation of STAT1, STAT3 and NFκB, since they secrete high levels of many inflammatory cytokines [36]. As preliminary evidence that macrophage abundance increases with age, we found that many transcripts specifically expressed in monocytes and macrophages (e.g. *CD14*, *CD163*) increase expression during kidney aging by analyzing microarray expression data from [1]. To examine how macrophage abundance changes with age *in vivo*, we performed immunohistochemistry using antibodies to CD163, a specific marker for macrophages [37]. We found a 2.1-fold higher abundance of interstitial macrophages in renal cortex sections from old versus young individuals (p = 0.006; Fig 5B).

To test whether individual variation in macrophage abundance correlates with variation in the activity of STAT1, STAT3 and NFκB, we first used the expression of three macrophage-specific transcripts (*CD163*, *CD14*, *TYROBP*) in the kidney DNA microarray data as an estimate of macrophage abundance in each renal cortex sample (see Methods). If macrophage infiltration contributes to the age-related increase in the activities of STAT1, STAT3 and NFκB, then one would expect that there would be a correlation between macrophage abundance and transcription factor activity in individual kidney samples, normalized for their ages. That is, among kidneys of the same age, those with higher relative macrophage abundance should also show higher levels of STAT1, STAT3 and NFκB activity, and vice versa. For each individual, we used linear regression to calculate whether that individual kidney sample showed high or low levels of macrophage-specific gene expression compared to the expected levels for their age. We then compared the estimated abundance of macrophages to the levels of activation of STAT1, STAT3 and NFκB in 73 individuals, adjusting for chronological age. We generated a heat map showing the estimated abundance of macrophages and transcription factor activity in the renal cortex, normalized for age. The heat map displays the correlation between estimated macrophage abundance with transcription factor activation as a dendrogram. We found that macrophage abundance was highly correlated with the activity of these three inflammatory transcription factors (r = 0.66–0.81, p < 10^−6^)(Fig 5A). The magnitude of this correlation suggests that macrophage abundance explains 40–65% of the variance in the activity of the three inflammatory transcription factors. In contrast, the expression of genes characteristically expressed in other immune cell types (i.e. T and B cells, natural killer cells, plasma cells, neutrophils) were not strongly correlated with the activity of STAT1, STAT3, NFκB, or with estimated macrophage abundance (S7 Fig).

To further examine the association between renal macrophage infiltration and activation of these transcription factors *in vivo*, we performed double-label immunofluorescence experiments to examine the correlation between interstitial macrophage abundance (CD163) and levels of nuclear pSTAT3 and pRelA in human kidney sections. We found that interstitial CD163^+^ macrophage infiltration was significantly correlated with pSTAT3 and pRelA nuclear immunoreactivity in epithelial compartments of the renal cortex (Fig 5C). Among the young kidney sections, the individual with the highest level of macrophage infiltration also had the highest level of STAT3 activation. Conversely, from the elderly, the individual kidney with the lowest burden of interstitial macrophages also had the lowest level of STAT3 activation (Fig 4C, r = 0.77, p < 0.001). We obtained similar associations between CD163^+^ macrophage abundance and NFκB activation (r = 0.83, p < 0.001)(Fig 5C). These results indicate that macrophage abundance is correlated with the activity of these inflammatory transcription factors in renal epithelial compartments, independent of chronological age.

### Macrophage-secreted factors induce age-associated transcriptional changes

Since activated macrophages are a source of inflammatory cytokines, we hypothesized that the transcriptional response evoked by macrophage-secreted factors might recapitulate gene expression changes associated with kidney aging [36]. To explore this, we analyzed gene expression changes in response to conditioned media from macrophages using data from two DNA microarray studies that characterized the transcriptional responses of human cells (endometrial stromal cells and adipocytes) to macrophage-conditioned media [38,39]. We identified a set of 77 genes that showed a concordant pattern of differential expression (p < 0.001 in both studies)(S7 Table) in response to macrophage-conditioned media, thereby defining a core gene expression signature for the response of human cells to macrophage-conditioned media. The transcriptional response to macrophage-conditioned media was strongly correlated with the gene expression changes that occur during kidney aging (r = 0.42, p < 10^−4^)(Fig 4D). This analysis indicates that macrophage-secreted signals are sufficient to evoke gene expression changes similar to those that occur during kidney aging.

### Genetic variants in NFκB genes associate with kidney function and chronic kidney disease

**Fig 6:**
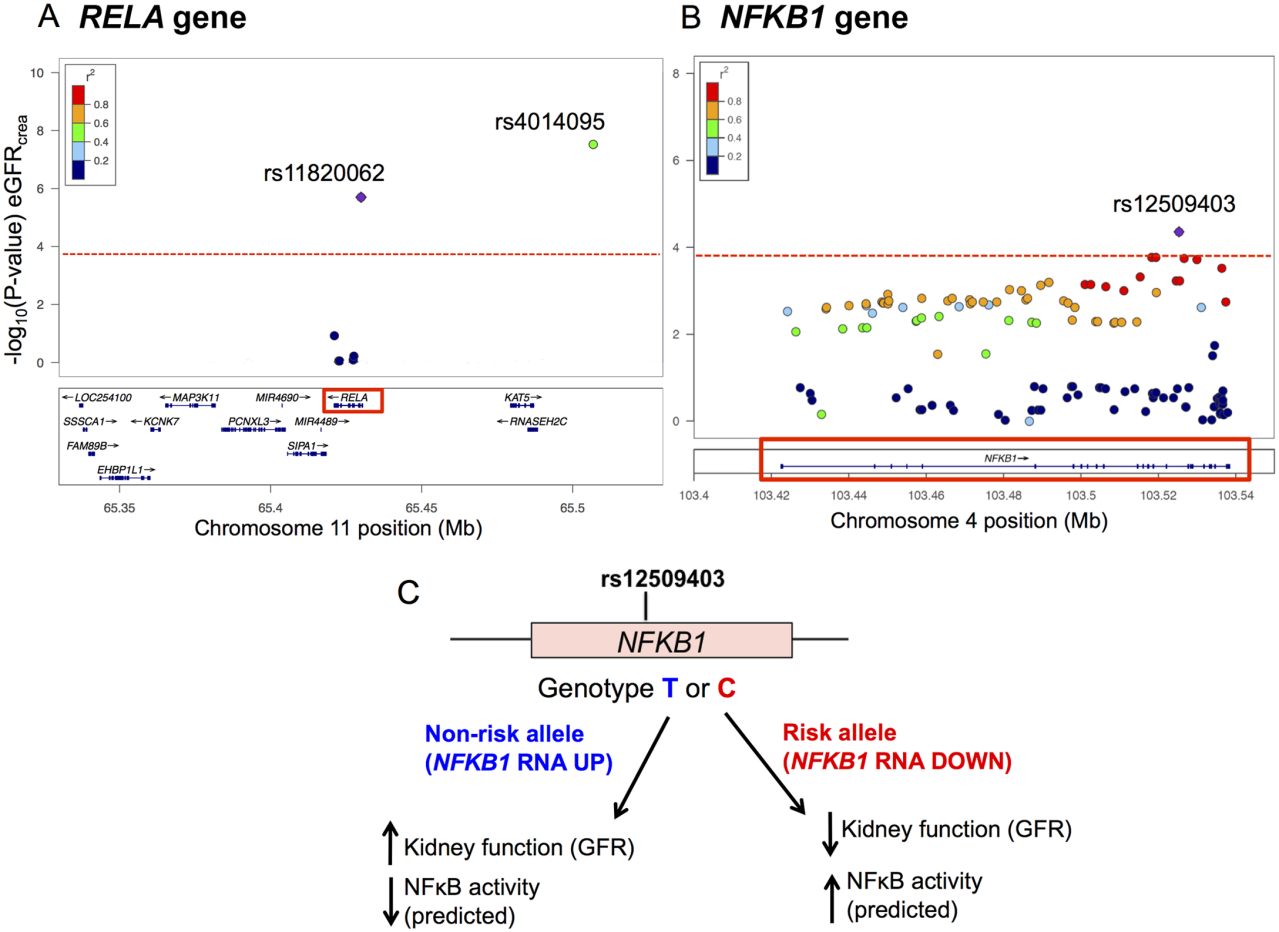
Genetic variation in NFκB genes associates with kidney function and chronic kidney disease. A. The regional association plot shows each SNP tested in the *RELA* gene arranged by its position on chromosome 11 (x-axis) with the–log_10_(p-value) for association with eGFR on the y-axis. The purple diamond represents the lead SNP within the RELA gene rs11820062. The colors of flanking SNPs in *RELA* represent their linkage disequilibrium (R^2^) with the lead SNP. The green dot represents rs4014195, an intergenic SNP that was previously associated with kidney function at genome-wide significance [40]. rs4014195 is moderately linked to rs11820062 in *RELA* (R^2^ = 0.44). Dotted line indicates Bonferroni significance level. B. The regional association plot shows each SNP tested in the *NFKB1* gene arranged by its position on chromosome 4 (x-axis) and the log_10_(p-value) for their association with eGFR on the y-axis. The purple diamond represents the lead SNP rs12509403. The color of dots representing flanking SNPs in the *NFKB1* gene indicates their linkage disequilibrium (R^2^) with the lead SNP as indicated in the heat map color key. Red dotted line indicates Bonferroni significance level. C. The schematic illustrates how rs12509403 genotype influences both *NFKB1* gene expression and kidney function. Rs12509403 correlates with *NFKB1* gene expression such that the T allele is associated with higher *NFKB1* mRNA expression and higher eGFR, while the C allele is associated with reduced mRNA expression and lower eGFR.

Given our observation that STAT1, STAT3 and NFκB contribute to the aging transcriptional program, we used the genes for these transcription factors as candidates to find out if they are associated with either kidney function or renal disease susceptibility. Specifically, we asked whether common single nucleotide polymorphisms (SNPs) in these transcription factor genes are associated with individual differences in estimated glomerular filtration rate (eGFR) or chronic kidney disease risk in the human population. We used publicly-available data from a large genome-wide association study of kidney function and chronic kidney disease that included 67,093 Caucasian individuals to test whether SNPs in *STAT1*, *STAT3*, or genes encoding the canonical NFκB transcription factor (*RELA* or *NFKB1*) are associated with either eGFR or chronic kidney disease [40]. We selected 217 SNPs within these four genes and queried the p-values for their associations with eGFR [40]. We identified lead SNPs in *NFKB1* (rs12509403) and *RELA* (rs11820062) that showed significant associations with eGFR after Bonferroni correction for multiple testing (Table 1 and Fig 6). The SNP rs11820062 in *RELA* also showed a significant association with chronic kidney disease susceptibility (OR = 1.088, p = 8.0 x 10^−5^)(Table 1).

**Table 1:** Genetic variation in NFκB genes associates with kidney function and chronic kidney disease.

SNP ID	Gene (location)	Major/minor allele^a^	Risk allele^a^	P-value (eGFR)^a^	P-value (CKD)^a^	Allelic Odds ratio^b^	eQTL? (risk allele)^c^
**rs12509403**	*NFKB1* (intron)	C/T	C	4.4 x 10^−5^	0.09	1.014	Reduced expression
**rs11820062**	*RELA* (intron)	C/T	C	2.0 x 10^−6^	8.0 x 10^−5^	1.088	No

^a^P-values for the association between genotype and estimated glomerular filtration rate (eGFR) or chronic kidney disease (CKD), major/minor alleles and minor allele frequencies are from [40]. These data were accessed from http://www.nhlbi.nih.gov/research/intramural/researchers/pi/fox-caroline/datasets.

^b^The estimated allelic odds ratios for chronic kidney disease were calculated using the association p-value, the sample size for cases and controls and minor allele frequency. Chi-squared statistics were used to match the association p-value with the distribution of minor allele carriers in chronic kidney disease cases and unaffected controls.

^c^ Indicates whether carrying the risk allele (defined as the allele correlated with reduced eGFR or with increased prevalence of CKD) correlates with elevated or reduced expression of its associated gene. eQTL data were published in [58] and accessed from http://genenetwork.nl/bloodeqtlbrowser/.

rs11820062 in *RELA* is moderately linked to rs4014195 (R^2^ = 0.44), an intergenic SNP that is located approximately 80 kilobases upstream of the *RELA* transcription start site (Fig 6A). rs4014195 has been previously associated with eGFR (p = 3 x 10^−8^) at genome-wide level statistical significance [40]. One possibility is that the phenotypic effect of rs4014195 on kidney function is mediated by changes in RelA expression. Alternatively, the phenotypic effect of rs4014195 might be due to changes in the activity of another nearby gene or several genes in its LD region.

Neither rs12509403 in *NFKB1* nor rs11820062 in *RELA* are linked to common SNPs that cause protein-coding changes. To ask whether these genetic variants may result in gene expression changes, we queried a large public dataset of expression quantitative trait loci (eQTLs) in peripheral blood [41]. We found that rs12509403 in *NFKB1* is associated with significant differences in mRNA expression of *NFKB1* (p = 1.03 x 10^−7^). Specifically, the C allele is associated with lower *NFKB1* expression, while the T allele is associated with higher *NFKB1* expression. The lower expression rs11820062 (C) allele is associated with a lower eGFR. This effect of the risk allele in *NFKB1* (encoding p50) is consistent with its known role in antagonizing the activity of the NFκB complex. The p50 subunit is found either as part of the RelA-p50 NFκB heterodimer or in p50-p50 homodimers. The p50-p50 homodimer binds and represses NFκB target genes by preventing their activation by RelA-containing complexes [18,19]. The genetic association result for *NFKB1* in humans shows a weak effect in the same direction as the null phenotype in mice; specifically, the human low-expressing *NFKB1* allele is associated with decreased kidney function and the *NFKB1* knockout phenotype in mice is characterized by increased RelA target gene expression and higher levels of chronic inflammation [42].

We examined SNPs linked to rs12509403 in order to identify candidates that might account for the variation in gene expression in *NFKB1*. For every SNP that is strongly linked to the lead SNP rs12509403 (R^2^ > 0.8), we examined data from ENCODE and asked whether the SNP resides within an H3K4me1 peak (often marks promoters), H3K27 acetylation peak (often marks active enhancers), a DNase I hypersensitivity cluster (marks accessible chromatin) or affects binding to a specific transcription factor. rs4640855 is linked to rs12509403 and falls within a ChIP-seq binding site for the Fos transcription factor, a DNase I hypersensitivity peak, and a H3K4me1 peak (S8 Fig and S8 Table). rs1598859 and rs3774963 are also linked to rs12509403 and fall within a DNase I hypersensitivity peak, an H3K4me1 peak and an H3K27 acetylation peak (S8 Fig and S8 Table). These three SNPs are candidates for being responsible for changes in the expression level of *NFKB1*.

## Discussion

### Inflammation is a dominant molecular signature of human kidney aging

We performed a high-throughput search for transcription factors that control gene expression changes during kidney aging. Out of 161 transcription factors screened, the top three hits from our analysis were the inflammation-associated transcription factors STAT1, STAT3 and NFκB, suggesting that they play a relatively important role in regulating age-associated gene expression changes in the kidney. We showed that STAT1, STAT3 and NFκB increase with age in the kidney, and that these increases can partially recapitulate the kidney aging transcriptome. A common feature of these transcription factors is that they mediate inflammatory responses. Thus, our results suggest a model in which the activity of STAT1, STAT3 and NFκB increase with age in the kidney, leading to transcriptional cascades and chronic inflammation in old age.

The regions of the kidney where STAT1, STAT3 and NFκB become activated in old age provide clues as to how these transcription factors are related to specific renal aging phenotypes. STAT1 was most strongly activated along Bowman’s capsule epithelial cells and in some cells along the periphery of glomeruli, a region where small capillaries become scarred in older kidneys (glomerulosclerosis). STAT3 activity was increased in glomerular cells, subsets of tubules and interstitial stromal cells in old age. Finally, NFκB was activated in most cells of glomerular and tubulointerstitial compartments in older renal cortex tissues. In summary, the regions showing activation of STAT1, STAT3 and NFκB during aging suggests a possible role for these transcription factors in the progression of glomerulosclerosis and renal interstitial fibrosis during aging.

### Effects of STAT1, STAT3 and NFκB activation on the kidney aging gene expression pattern

We showed that activation of STAT1, STAT3 and NFκB by inflammatory cytokines in HK-2 cells caused transcriptional changes in their direct targets that are similar to their expression changes during kidney aging. We restricted our analysis to direct transcription factor targets in order to capture the primary effects of these transcription factors. It is important to note that STAT1 and STAT3 share several of the same direct targets (e.g. *STAT3*, *BCL6*, *NNMT*, *TNFRSF1A*). Moreover, STAT1 and STAT3 can compete for binding at the same DNA sequence motifs, or they can regulate target gene expression as STAT1-STAT3 heterodimers [43]. Hence, there may be redundancy or cooperativity in the roles of STAT1 and STAT3 in regulating transcriptional changes during kidney aging.

By activating STAT1, STAT3 and NFκB simultaneously, we were able to cause transcriptional changes that recapitulated a significant portion of the kidney aging expression profile. Specifically, the correlation between the transcriptional changes caused by activation of all three transcription factors together and aging was r = 0.51 (p < 10^−5^). The magnitude of this correlation, as well as the strong ChIP-seq binding enrichments for these transcription factors at kidney age-related genes indicates that the combined effects of STAT1, STAT3 and NFκB target gene activation play an important role in regulating gene expression changes in the aging kidney.

### Functional consequences of STAT1, STAT3 and NFκB activation

Is increased activity of STAT1, STAT3 and NFκB detrimental to the pathophysiology of older kidneys? Several studies suggest that increased activity of JAK/STAT and NFκB transcription factors can have deleterious effects on the kidney in response to renal injury or inflammatory renal disease. In mice with lupus nephritis (an inflammatory kidney disease), STAT1 becomes activated and blockade of STAT1 activity reduces macrophage infiltration and improves renal function [44,45]. Similarly, in a mouse model of obstructive kidney injury, STAT3 is activated and inhibition of STAT3 with the drug S3I-201 attenuates interstitial fibrosis and immune cell infiltration in the injured kidney [46]. In a mouse model of diabetic kidney disease (the most common form of human chronic kidney disease), mice that are heterozygous for a STAT3 loss of function mutation have reduced interstitial fibrosis and inflammation, and improved renal function compared to diabetic mice with wild-type STAT3 [47]. Other studies have reported that inhibition of NFκB activity has a protective effect in rodent models of renal injury and renal aging [48,49]. While most of these studies were not performed in the context of aging, these rodent models of renal injury and disease recapitulate many of the histopathologic features of human kidney aging, such as interstitial fibrosis and inflammation. Therefore, the results from the renal disease models suggest that elevated activity of STAT1, STAT3 and NFκB may have a deleterious effect on kidney physiology and function in old age. Moreover, previous work has shown that NFκB plays a multifaceted role in promoting aging-related changes in several tissues [6,42,50–52].

### Possible upstream activators of STAT1, STAT3 and NFκB in old age: Inflammatory cytokines and macrophages

The inflammatory cytokines IFNγ, IL-6 and TNFα are upstream activators of STAT1, STAT3 and NFκB, respectively. The systemic circulating levels of IL-6 and TNFα increase with age and the production of IFNγ by subsets of lymphocytes is increased in elderly individuals [53–55]. These observations suggest that these three cytokines may be partially responsible for increased activity of STAT1, STAT3 and NFκB in the kidney in old age. However, whether IFNγ, IL-6 or TNFα are the main factors responsible for activating STAT1, STAT3 and NFκB *in vivo* during kidney aging remains unclear, since other signals can also activate these three transcription factors. For example STAT1 can be activated by type I interferon, STAT3 can be activated by growth factors (e.g. EGF, LIF) and NFκB can be activated by reactive oxygen species, infection and other inflammatory cytokines, including IL-1β [18,19]. Further studies *in vivo* should illuminate which of these upstream signals are responsible for activation of STAT1, STAT3 and NFκB in the aging kidney.

Macrophages are known to secrete a variety of inflammatory cytokines, including IL-6, IFNγ and TNFα [36]. Three observations suggest that renal macrophage infiltration might contribute to the activation of STAT1, STAT3 and NFκB in the kidney. First, macrophage infiltration increases with age in the human kidney. Second, the general transcriptional response of human cell lines to macrophage-conditioned media recapitulates gene expression changes that occur during kidney aging. Third, variation in the activity of STAT1, STAT3 and NFκB are correlated with variation in macrophage abundance between individuals, independent of chronological age. This correlation suggests that there is a common underlying mechanism linking increased macrophage infiltration with the activity of these three transcription factors in the kidney epithelium, or that macrophages themselves may potentiate the activation of STAT1, STAT3 and NFκB in the renal parenchyma.

Nevertheless, it remains unclear whether macrophage infiltration is the primary contributor to inflammatory gene expression changes in the epithelial compartments during kidney aging, and whether macrophage infiltration contributes to functional decline of the kidney. In mice, blocking macrophage infiltration via inhibition of CCR2 (the receptor for CCL2/monocyte chemotactic protein-1) reduces renal fibrosis and inflammation and improves renal function in diabetic kidney disease, suggesting that infiltrating macrophages might play a causative role in the progression of renal fibrosis [56]. In addition to macrophages, other tissues and cell types have been suggested to promote chronic inflammation in the aging kidney, including senescent cells and peripheral adipose tissue [57].

### A novel genetic association between NFκB and kidney function

Since STAT1, STAT3 and NFκB regulate gene expression changes during aging, we wanted to examine the functional role of these transcription factors in the human kidney. That is, does genetic variation in the genes encoding these transcription factors influence kidney function or chronic kidney disease susceptibility? Glomerular filtration rate varies considerably in individuals of the same age. In old age, some individuals maintain relatively high levels of kidney function, while others suffer from chronic kidney disease [3].

We used data from a large genome-wide association study [40] to examine whether common SNPs in any of the genes encoding the kidney aging transcription factors (STAT1, STAT3 and the canonical NFκB complex genes *RELA* and *NFKB1*) show an association with kidney function or chronic kidney disease. We found two independent DNA variants in the NFκB transcription factor genes *RELA* and *NFKB1* (rs11820062 and rs12509403, respectively) that associate with kidney function and chronic kidney disease susceptibility in the human population. rs12509403 in *NFKB1* is also associated with variation in *NFKB1* gene expression [58], suggesting that variation in expression of *NFKB1* may explain the phenotypic effect of this SNP on kidney function. Our findings provide genetic evidence that variation in the genes encoding the canonical NFκB transcription factor are associated with differences in kidney function and chronic kidney disease susceptibility in the human population. Furthermore, the genetic association between NFκB and age-related renal phenotypes suggests that the observed increase in NFκB activity during kidney aging may contribute to the age-related decline in renal function.

In this study, we identified three inflammation-induced transcription factors that drive transcriptional cascades in the aging kidney. Our work adds to a growing body of evidence that chronic inflammation is a contributing factor to aging phenotypes in human tissues. Future studies should provide additional insights about how STAT1, STAT3 and NFκB affect renal function and age-related phenotypes *in vivo*, as well as the primary upstream signals responsible for their activation in the aging kidney.

## Materials and Methods

### Identification of ENCODE transcription factors that bind kidney age-related genes

961 ChIP-seq data sets for 161 transcription factors (on human genome version hg19) were obtained from the ENCODE Consortium as of 07/13/12 (http://genome.ucsc.edu/ENCODE/downloads.html). All binding sites with q-value or p-value < 10^−5^ were considered, and ChIP-seq peaks less than 20 base pairs long were discarded. Binding sites were then associated with ENSEMBL-annotated hg19 transcripts (ensGene, downloaded 12/9/13) if the position of maximum read density within the binding site was located within 5 kb upstream or 2 kb downstream of the annotated transcription start site.

Prior work has shown that a parameter termed complexity affects whether or not a ChIP-seq binding peak for a specific transcription factor is likely to identify a gene whose expression is responsive to knockdown of that transcription factor [10]. For most transcription factors, binding sites vary from low complexity sites (those bound by a small number of transcription factors) to high complexity sites (those bound by many transcription factors). Sites bound by a large number of transcription factors are not generally responsive to changes in just one transcription factor. We calculated the complexity of every ChIP-seq binding site, defined as the number of transcription factors that were found to have a significant binding site that overlaps that region. Overlapping binding sites for the same transcription factor observed in multiple cell lines or replicate experiments were only counted once.

We performed a pilot analysis to investigate how binding complexity might affect the selection of transcription factors that bind to the age-related kidney genes. The list of kidney age-related genes was obtained from supplemental data published in [1], which originally identified age-regulated probesets corresponding to 630 genes used for the analysis in this paper. We compared the overlap between ChIP-seq binding peaks and age-related kidney genes using all of the binding peaks, or only the binding peaks with <50% complexity (i.e. bound by 81 or fewer transcription factors). To focus our analysis on transcription factors that act within the renal parenchyma, we selected transcription factors that showed detectable expression in epithelial compartments using IHC data from the human protein atlas. We compared the degree of overlap between the list of ChIP-seq targets to the list of age-regulated genes.

The results for the screen using the binding sites with <50% complexity is shown in Fig 1B, and the results using all of the transcription factor binding sites are shown in S9 Fig. The two screens returned similar lists of transcription factors enriched for binding to age-related kidney genes; specifically, STAT1, NFκB and STAT3 were identified consistently. For this paper, we selected transcription factor binding targets with a binding complexity <50%.

For enrichment calculations, the set of all genes present on the U133A and B microarrays were used as a background set. Significance of overlap was determined by Fisher’s Exact test, with Chi-Square approximation where appropriate (all values greater than 5).

To restrict our analysis to *bona fide* transcription factors, we excluded non-specific DNA-binding machinery, such as factors associated with RNA polymerase II and components of basal transcriptional machinery (e.g. p300, CTCF, RAD21, TAF1). Finally, we selected only transcription factors that showed detectable staining in epithelial compartments of the kidney (tubules or glomeruli) in IHC experiments generated by the human protein atlas (http://www.proteinatlas.org/). We thereby removed transcription factors that are specifically expressed in immune cells (i.e. *PU1*, *EBF1*, *IKZF1*, *BCL11A*, *BCLAF1*), which would otherwise appear enriched in our screen. Transcription factor ChIP-seq experiments with a Chi-square or Fisher’s exact p-value of less than 5 x 10^−5^ (significant after Bonferroni correction for all ChIP-seq experiments tested) and a >1.5 fold enrichment were considered statistically significant.

### Immunohistochemistry

We obtained paraffin-embedded normal human kidney samples from the Stanford Department of Surgical Pathology. Samples were extracted from tumor-free normal tissue in patients who underwent nephrectomy for renal tumors. Tissues were sectioned onto slides for hematoxylin and eosin staining and immunostaining experiments. Slides containing sections of paraffin-embedded kidney tissues were deparaffinized in three changes of xylene. Antigen retrieval was performed in a pressure cooker using either tris-EDTA (pH 9.0) for pSTAT3, pRelA and CD163 antibodies or in citrate buffer (pH 6.0) for STAT3 and NFκB p65/RelA antibodies. Blocking and washing steps were performed according to the manufacturer’s protocol using the Expose detection IHC kit (Abcam, Cambridge, MA). The primary antibodies used were rabbit polyclonal STAT3 (1:400, Cell Signaling, Danvers, MA), rabbit monoclonal to phospho^Tyr705^ STAT3 (1:20, Cell Signaling), rabbit polyclonal NFκB p65/RelA (1:80, Cell Signaling), rabbit polyclonal phospho^Ser536^ NFκB p65/RelA (1:40, Novus Biologicals, Littleton, CO), rabbit polyclonal to STAT1 (1:80, Abcam), rabbit polyclonal pSTAT^Tyr701^ (1:60, Sigma-Aldrich, St. Louis, MO). The appropriate horseradish peroxidase-conjugated secondary antibodies were used for chromogenic signal detection with 1,2-diaminobenzidene in buffered substrate (Expose detection IHC kit, Abcam). Slides were counterstained with hematoxylin. Semi-quantitative scoring of IHC stains was performed blinded to chronological age, using a 0–5 point relative scoring scheme. Scores reflect both the signal intensity and the percentage of positively-stained cells from three renal cortical fields at 20X magnification, similar to previously described composite scoring schemes [59,60]. For pSTAT1, pSTAT3 and pRelA, only nuclear staining was scored as a marker for transcription factor activation. Statistical differences in IHC staining scores between the young and old renal cortex tissues were calculated using the Mann-Whitney U test (one-tailed), testing the hypothesis that transcription factor activity increases with age.

### Double-label immunofluorescence

For double-label immunofluorescence staining of paraffin-embedded tissue sections, slides were blocked for 1 hour in blocking buffer (5% bovine serum albumin/0.5% Triton in PBS), rinsed in PBS and incubated overnight at 4°C in primary antibodies. For co-immunofluorescence staining of HK-2 cells, cells were fixed in 4% paraformaldehyde and blocked for 1 hour in blocking buffer (5% bovine serum albumin/0.5% Triton in PBS), rinsed in PBS, and incubated for 1 hour in primary antibodies at room temperature. Primary antibodies used were mouse monoclonal anti-vimentin (Abcam) and rabbit polyclonal phospho^Ser536^ NFκB p65/RelA (1:40, Novus Biologicals), rabbit monoclonal phospho^Tyr705^ STAT3 (1:30, Cell signaling) and mouse anti-CD163 (1:40, Novus biologicals) and rabbit anti-CD163 (1:40, Novus Biologicals). Slides were washed in PBS and co-incubated with the appropriate AlexaFluor conjugated secondary antibodies (1:500, anti-rabbit Alexa 488 and 1:500 anti-mouse Alexa 647, Abcam) in the dark for 1 hour. Slides were mounted with Vectashield DAPI mounting medium (Vector Labs, Burlingame, CA) to label DNA and imaged on a Zeiss Axioplan fluorescence microscope (Oberkochen, Germany). Staining was quantified on ImageJ by counting the number of cells (or DAPI nuclei for the transcription factors) that stained positive for the indicated antibodies on at least three renal cortical fields at 20X magnification.

### Cell culture experiments

Human renal proximal tubular epithelial cells immortalized with human papillomavirus 16 (HK-2 cells) were maintained in keratinocyte-serum free medium (ATCC; Life Technologies, Carlsbad, CA) supplemented with bovine pituitary extract, human recombinant epidermal growth factor and 1% penicillin-streptomycin (Life Technologies). For all cytokine treatment experiments, cells were split into six-well plates and grown to 70% confluence. The cytokines used were recombinant human IL-6 (Cell Signaling Technologies), recombinant human TNFα (Cell Signaling Technologies) and recombinant human IFNγ (Cell Signaling Technologies). We used the drug S3I-201 (Selleck Chemicals) for STAT3 inhibition. For STAT3 inhibition experiments, cells received 50 uM of S3I-201 pre-treatment for 1 hour, followed by addition of 200 ng/mL human recombinant IL-6 for an additional 90 minutes. For TNFα and IFNγ stimulation experiments, cells were incubated with 100 ng/mL of recombinant TNFα or IFNγ respectively, or PBS vehicle control treatment.

### Microarray expression profiling experiments and associations with kidney aging

RNA was isolated at 90 minutes post-cytokine treatment using TRIZOL reagent (Life Technologies) according to the manufacturer’s protocol. Total RNA samples were submitted to the Stanford functional genomics DNA microarray core facility for assessment of RNA integrity. Each RNA sample was amplified using the Ambion Illumina RNA amplification kit with biotin UTP labeling. The Ambion Illumina RNA amplification kit uses T7 oligo(dT) primer to generate single stranded cDNA followed by a second strand synthesis to generate double-stranded cDNA, which was then column purified. *In vitro* transcription was performed to synthesize biotin-labeled cRNA using T7 RNA polymerase, and cRNA was column purified. The cRNA was then measured and total of 750 ng of cRNA was hybridized to array using standard Illumina protocols with streptavidin-Cy3 being used for signal detection. Slides were scanned on an Illumina Beadstation and analyzed using BeadStudio (Illumina, Inc). Microarray expression data were normalized using the rank invariant normalization method [61]. Significant differential gene expression was determined using an unpaired Student’s t test for treated vs. control samples, and p-value thresholds used for statistical significance are indicated in each specific analysis, as appropriate. Only probe sets with detectable expression (signal detection p < 0.01 in at least one experimental sample per batch of microarray experiments) were analyzed for differential expression in subsequent analyses. For genes represented by multiple Illumina probe sets, the most differentially expressed probe set was used in subsequent analyses. Heat maps showing comparative gene expression patterns between different cytokine/transcription factor perturbations in HK-2 cells and in kidney aging were generated using MultiExperiment Viewer (MeV version 4.0) software (http://www.tm4.org/). Direct transcription factor targets for STAT1, STAT3 and NFκB were defined as the subset of high-confidence ENCODE ChIP-seq targets from the proximal-filtered list published in [11] that also showed differential expression (p < 0.05 in microarray experiments) following the appropriate cytokine stimulation. For analysis of cytokine-regulated genes (S3 Fig), we surveyed the entire transcriptome (9,508–10,121 detectably expressed probe sets) for changes in gene expression, rather than restricting our analysis to the ChIP-seq binding targets for the three transcription factors (91 to 134 ChIP-seq binding targets). Therefore, we used a more stringent statistical threshold (p < 0.001) for the identification of cytokine-regulated genes. Similarities between gene expression profiles were determined by the Pearson correlation between gene expression changes following inflammatory cytokine stimulation (log_2_fold-change) vs. gene expression changes during kidney aging (age-related slope/beta coefficient); the p-value for the concordance in expression pattern (percentage of expression changes in a concordant direction) between indicated gene sets was calculated using a binomial test. All microarray datasets generated in this study are publicly available in the Gene Expression Omnibus (GEO Accession numbers: GSE68940, GSE68941, GSE68942, GSE68826).

### Correlations between STAT1, STAT3 and NFκB activity and macrophage markers

To assess the correlations between STAT1, STAT3 and NFκB targets with macrophage markers, we analyzed published microarray data from [1]. STAT3 targets were defined as STAT3 ChIP-seq targets [11] that were also induced by IL-6 (at a significance level of p *<* 0.05) from our microarray analysis. Similarly NFκB and STAT1 direct targets were defined as ChIP-seq targets that were induced by TNFα or IFNγ (p < 0.05) and non-overlapping with the set of targets for the other two transcription factors. For each transcription target gene, we converted its normalized expression values into normalized z-scores across the 73 individual renal cortex samples and computed the averaged expression z-score. We then performed a linear regression analysis of average expression z-scores vs. chronological age to generate a linear regression equation for expression z-score vs. age. The averaged expression z-scores were converted to age-adjusted expression values based on the linear relationship between age and expression. Similarly, for transcripts specific to (or strongly enriched in) monocytes/macrophages (e.g. *CD163*, *CD14*, *TYROBP*), each expression value was converted into a z-score, averaged and regressed on age to derive an age-adjusted expression level. Heat maps showing age-adjusted expression values for macrophage transcripts and transcription factor target genes were generated using MeV version 4.0 software (Boston, MA). Rows and columns were hierarchically clustered by Pearson correlation.

### Genetic association study for kidney function and chronic kidney disease

SNPs within the STAT1, STAT3 and canonical NFκB transcription factor genes (*RELA* and *NFKB1*) were selected using SCANdb (http://www.scandb.org/newinterface/index.html) and intersected with the list of all imputed SNPs from a previously published genome-wide association study of kidney function and chronic kidney disease [40,62]. In total, we tested 217 SNPs for their association with kidney function and chronic kidney disease. Thus, SNPs with an association p-value of less than 1.1 x 10^−4^ (0.05/434 tests) remained statistically significant after Bonferroni correction for multiple testing. LocusZoom (http://csg.sph.umich.edu/locuszoom/) was used to generate regional association plots for *RELA* and *NFKB1*, illustrating the statistical association between each of the tested SNPs in *RELA* and *NFKB1* with eGFR.

## Supporting Information

S1 Fig Including renal function as a covariate in the linear regression reduces the age-coefficients for kidney age-related genes. Each point on the scatterplot is an age-related gene in the renal cortex from [1]. The x-axis shows the age-coefficient for these age-related genes when GFR is a covariate in the linear regression model. The y-axis shows the age-coefficient for these age-related genes when GFR is not a covariate in the model. Adding GFR as a covariate shifts most of the genes to the left of the y = x line (in red), indicating that expression of most of the age-related genes is informative of renal function, independently of age.
(TIF)

S2 Fig Comparison of the kidney aging transcriptional profile between humans and rats. The heat map shows expression changes for 427 human kidney age-related genes and their orthologous genes in rats from a microarray study of rat kidney aging [14](GSE47070). The left column of the heat map shows the age-slopes for these genes in humans (from Rodwell et al. 2004) and the right column of the heat map shows the log_2_ fold-change in expression between old (104 week old) and young (21 week old) rats. The overall kidney aging transcriptional profiles in humans and rats are highly correlated (r = 0.46, p < 10^−5^).
(TIF)

S3 Fig Response of HK-2 cells to the inflammatory cytokines IL-6, TNFα and IFNγ recapitulates kidney aging at the transcriptional level. A. Comparison of 48 IFNγ-regulated genes (p < 0.001) following IFNγ-stimulation in HK-2 cells with their expression profile during kidney aging. B. Comparison of 32 IL-6-regulated genes in HK-2 cells (p < 0.001) with their expression profile during kidney aging. C. Comparison of 230 TNFα-regulated genes in HK-2 cells (p < 0.001) with their expression profile during kidney aging. The left column of the heat maps shows the log_2_ fold-changes of these transcripts following cytokine stimulation and the right column of the heat map shows the corresponding log_2_-adjusted beta coefficient (age-slope) for these transcripts during kidney aging, using data from [1].
(TIF)

S4 Fig Limited overlap between STAT1, STAT3 and NFκB direct targets. The venn diagram shows the degree of overlap between the direct targets of STAT1, STAT3 and NFκB (as defined in Methods). Most of the direct targets for these three transcription factors are non-overlapping.
(TIF)

S5 Fig Induction of STAT1, STAT3 or NFκB direct targets by combinations of inflammatory cytokines recapitulates the kidney aging gene expression pattern in HK-2 cells. The first seven columns of the heat map shows gene expression changes in response to combinations of inflammatory cytokines. Each of the 74 rows corresponds to a gene that is a direct target of STAT1, STAT3, or NFκB. The color indicates log_2_ fold-changes in gene expression for the direct targets. The last column of the heat map shows the corresponding log_2_-adjusted beta coefficient (age-slope) for these transcripts during kidney aging, using data from Rodwell et al. 2004 [1].
(TIF)

S6 Fig The transcriptional response to the inflammatory cytokines IL-6, TNFα and IFNγ is a specific signature of kidney aging when compared to other cytokines/growth factors. The y-axis of the histogram shows the Pearson correlations between gene expression changes following stimulation of human cells with different cytokines and growth factors indicated on the x-axis and their expression changes during kidney aging. Genes with significant differential expression at p < 0.001 were considered significant in these analyses. Three of the datasets were from the present study (red bars). The remaining microarray datasets were downloaded from GEO [20–30], and include the responses of other cells to IL-6, TNFα and IFNγ as well as the responses of diverse human cell types to 10 other cytokines or growth factors (VEGFA, Wnt3a, PDGF, IL10, TGF-ß EGF, HGF, IL17, IL1ß, IFNα). *Indicates p < 0.05, **p < 0.005 (Pearson correlation p-value).
(TIF)

S7 Fig Co-association between estimated immune cell abundance for different leukocyte classes with the estimated activity of STAT1, STAT3 and NFκB. Each row in the heat map shows the estimated activity of STAT1, STAT3, NFκB (based on target gene expression) or the abundance of macrophage, B cells, T cells, NK cells, neutrophils, plasma cells (estimated by marker expression). Each column represents an individual kidney RNA sample. The color indicates the level of transcription factor activation or the abundance of cells in an individual kidney, normalized for age. The score shows whether the activity of the transcription factor or estimated abundance of immune cell types is higher or lower than the average for others at the same age. Red color indicates individuals with high relative expression or cell abundance and blue color indicates individuals with low relative expression or cell abundance compared to the age-adjusted mean. The dendrogram on the left shows that correlation of transcription factor activity is higher with estimated macrophage abundance than with the estimated abundance of other types of immune cells.
(TIF)

S8 Fig SNPs in the LD region of the NFKB1 lead SNP rs12509403 are associated with open chromatin and potential regulatory elements. UCSC genome browser screen shot showing the *NFKB1* gene (exons shown as boxes). The grey box indicates a Fos ChIP-seq binding peak that contains rs4648055. Shown below are tracks for H3K4me1 (a mark found near regulatory elements) and H3K27ac (a common mark of active enhancers). The colors of the peaks represent different cell lines. The grey and black boxes indicate Dnase I hypersensitivity clusters in the lower panel of the image. rs12509403 is the lead eQTL SNP. Shown are three SNPs linked to rs12509403 (R^2^ > 0.8) that may affect expression of *NFKB1* as they occur within Dnase I hypersensitivity clusters, regulatory histone marks, or affect transcription factor binding.
(TIF)

S9 Fig STAT1, STAT3 and NFκB remain significantly enriched for binding kidney age-related genes when high complexity binding sites are included. We repeated the screen of the ENCODE ChIP seq database using all transcription factor binding peaks, rather than only using binding peaks with a complexity score <81 as in Fig 1B. Top. The histogram shows the results for the three transcription factors that meet the selection criteria used in Fig 1B: enrichment p-value that is Bonferroni significant (p < 5 x 10^−5^) and a >1.5-fold enrichment. Bottom. Table shows the top four ChIP-seq datasets for enrichment for binding to kidney age-related genes along with the transcription factor and cell lines used, ranked by p-value. STAT1, STAT3 and NFκB are among the top four ChIP-seq datasets that show enrichment for binding to the kidney age-related genes.
(TIF)

S1 Table Summary data for STAT1, STAT3 and NFκB ChIP-seq enrichments for kidney age-related genes in different cell lines. (XLS)

S2 Table Summary data for overlaps between differentially regulated genes and ENCODE transcription factor binding profiles using random microarray datasets from GEO. (XLS)

S3 Table List of 114 kidney age-related genes in common between human and rat. (XLS)

S4 Table Top five ENCODE transcription factor overlaps for 114 shared kidney age-related genes in human and rat. (XLS)

S5 Table Cytokine-responsive genes and direct targets of STAT1, STAT3 and NFκB from ChIP-seq datasets and microarray expression profiling. (XLS)

S6 Table Microarray summary data for selected genes related to epithelial-mesenchymal transition and collagen genes following TNFα stimulation of HK-2 cells. (XLS)

S7 Table List of macrophage conditioned media responsive-genes. (XLS)

S8 Table Description of ENCODE functional annotations for SNPs in the linkage disequilibrium region of rs12509403 in the *NFKB1* gene. (XLS)
