# Supplementary figures and images for: The Inflammatory Transcription Factors NFκB, STAT1 and STAT3 Drive Age-Associated Transcriptional Changes in the Human Kidney
Source: PLoS Genet. 2015 Dec 17;11(12):e1005734. doi: 10.1371/journal.pgen.1005734 (PMC4682820; doi:10.1371/journal.pgen.1005734)

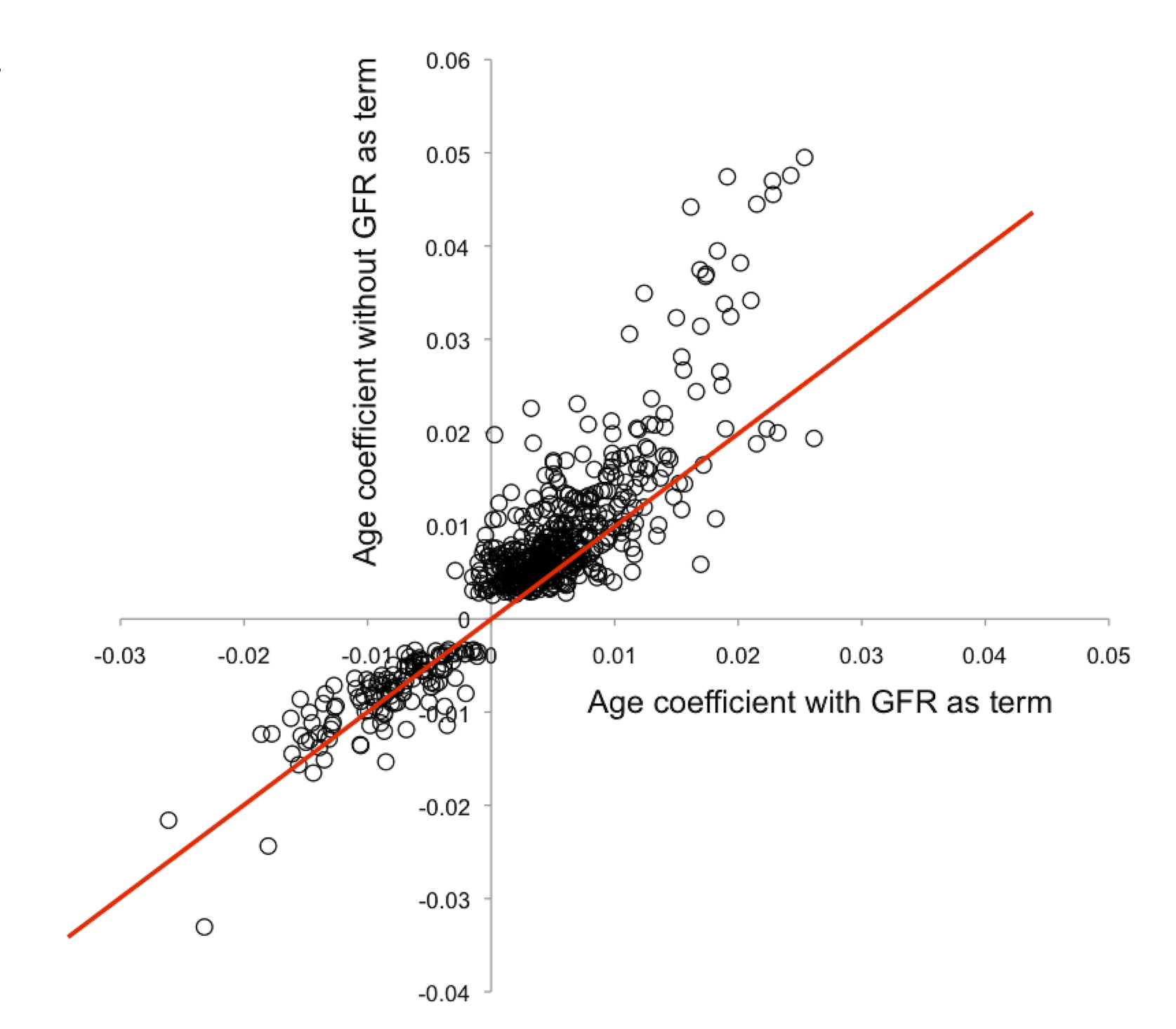

Supplement: S1 Fig — Each point on the scatterplot is an age-related gene in the renal cortex from [1]. The x-axis shows the age-coefficient for these age-related genes when GFR is a covariate in the linear regression model. The y-axis shows the age-coefficient for these age-related genes when GFR is not a covariate in the model. Adding GFR as a covariate shifts most of the genes to the left of the y = x line (in red), indicating that expression of most of the age-related genes is informative of renal function, independently of age. (TIF) [file pgen.1005734.s001.tif]

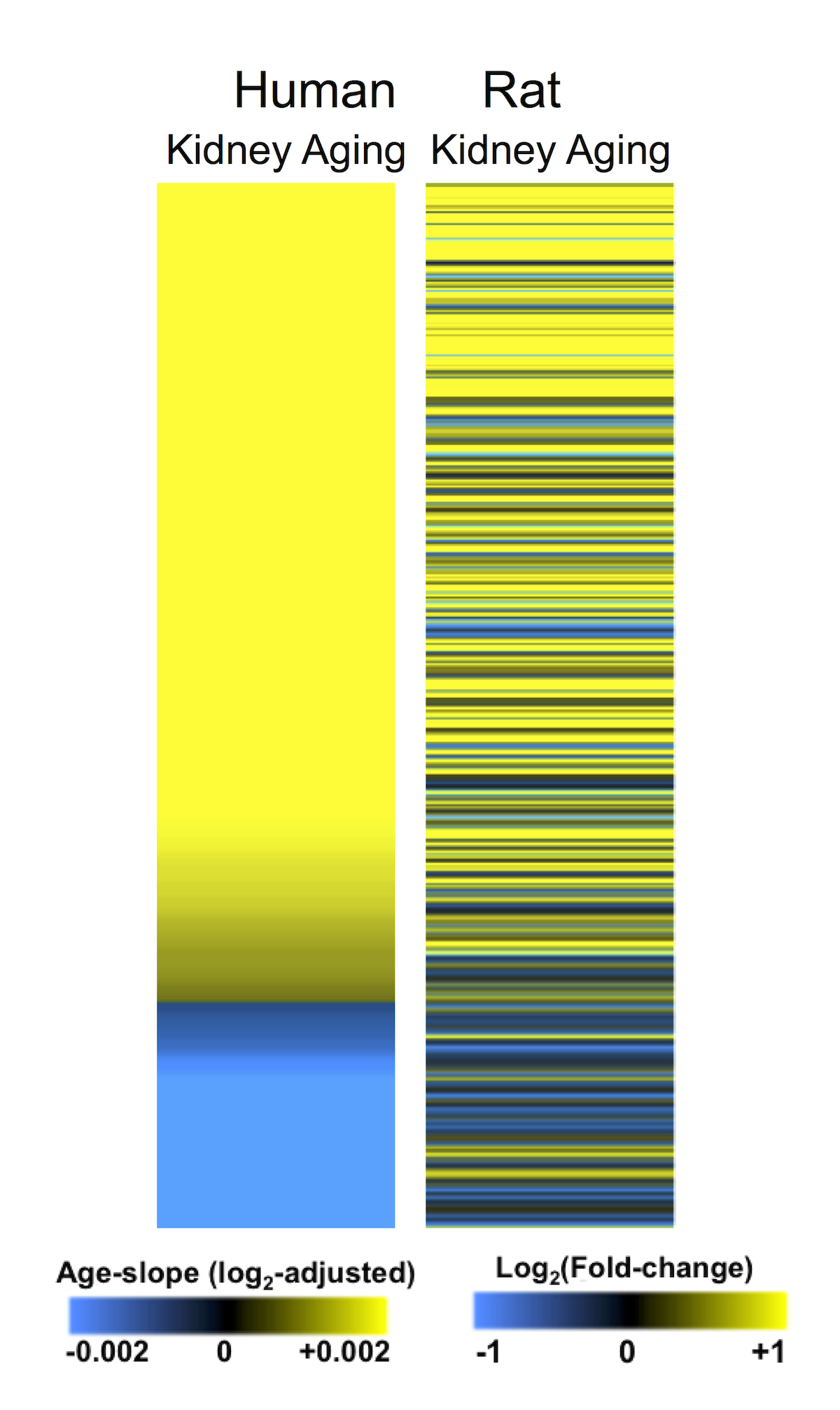

Supplement: S2 Fig — The heat map shows expression changes for 427 human kidney age-related genes and their orthologous genes in rats from a microarray study of rat kidney aging [14](GSE47070). The left column of the heat map shows the age-slopes for these genes in humans (from Rodwell et al. 2004) and the right column of the heat map shows the log2 fold-change in expression between old (104 week old) and young (21 week old) rats. The overall kidney aging transcriptional profiles in humans and rats are highly correlated (r = 0.46, p < 10−5). (TIF) [file pgen.1005734.s002.tif]

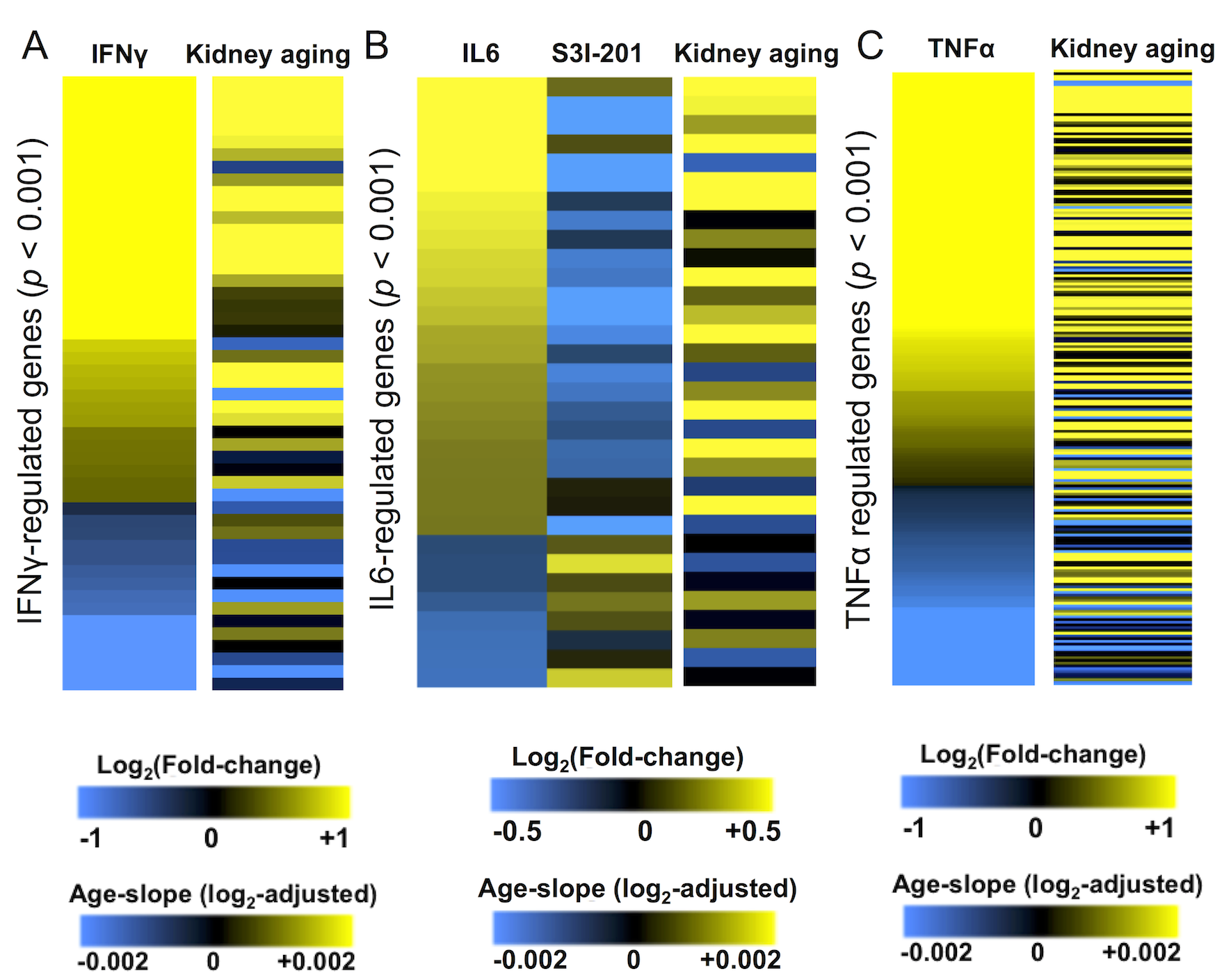

Supplement: S3 Fig — A. Comparison of 48 IFNγ-regulated genes (p < 0.001) following IFNγ-stimulation in HK-2 cells with their expression profile during kidney aging. B. Comparison of 32 IL-6-regulated genes in HK-2 cells (p < 0.001) with their expression profile during kidney aging. C. Comparison of 230 TNFα-regulated genes in HK-2 cells (p < 0.001) with their expression profile during kidney aging. The left column of the heat maps shows the log2 fold-changes of these transcripts following cytokine stimulation and the right column of the heat map shows the corresponding log2-adjusted beta coefficient (age-slope) for these transcripts during kidney aging, using data from [1]. (TIF) [file pgen.1005734.s003.tif]

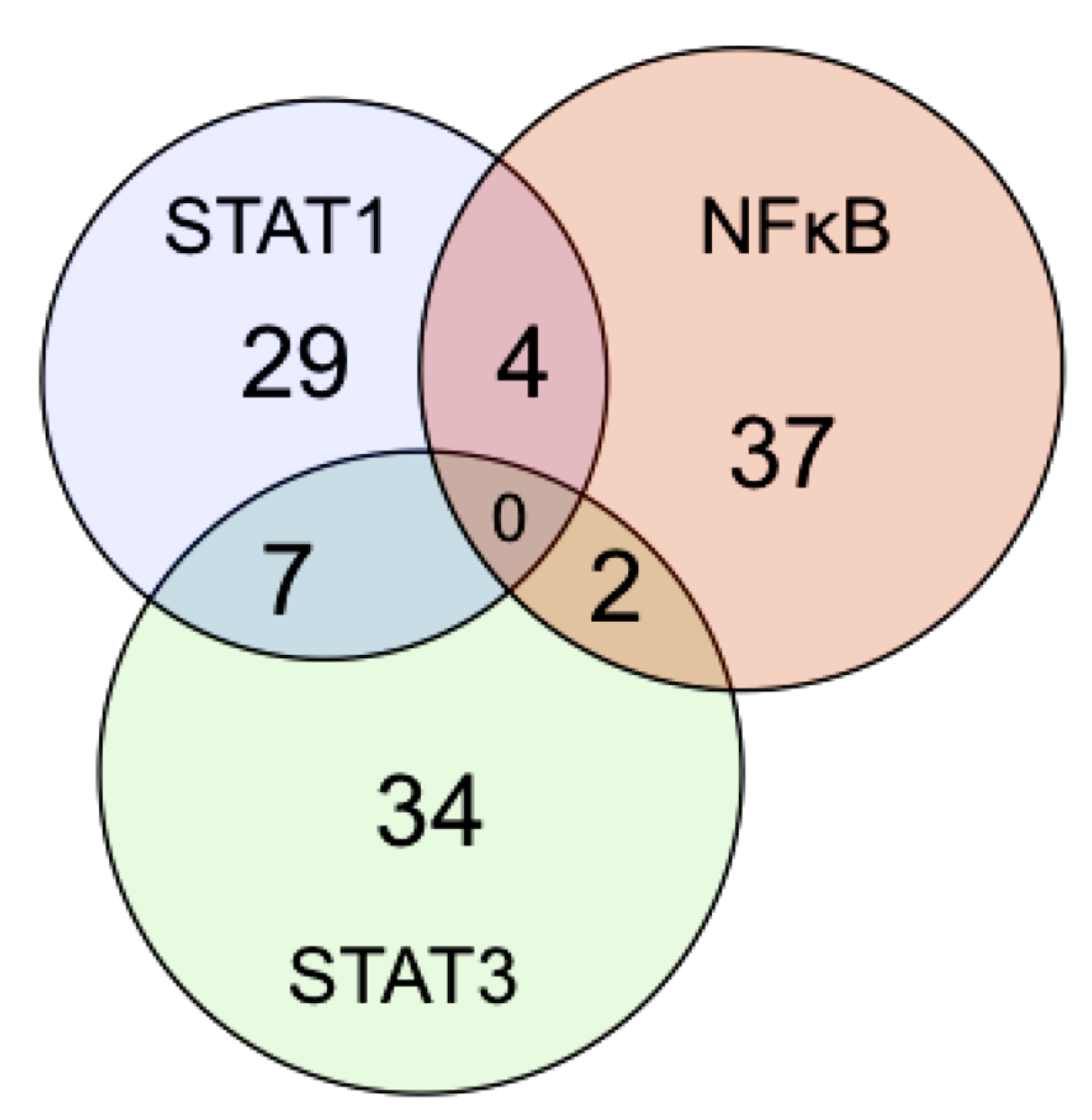

Supplement: S4 Fig — The venn diagram shows the degree of overlap between the direct targets of STAT1, STAT3 and NFκB (as defined in Methods). Most of the direct targets for these three transcription factors are non-overlapping. (TIF) [file pgen.1005734.s004.tif]

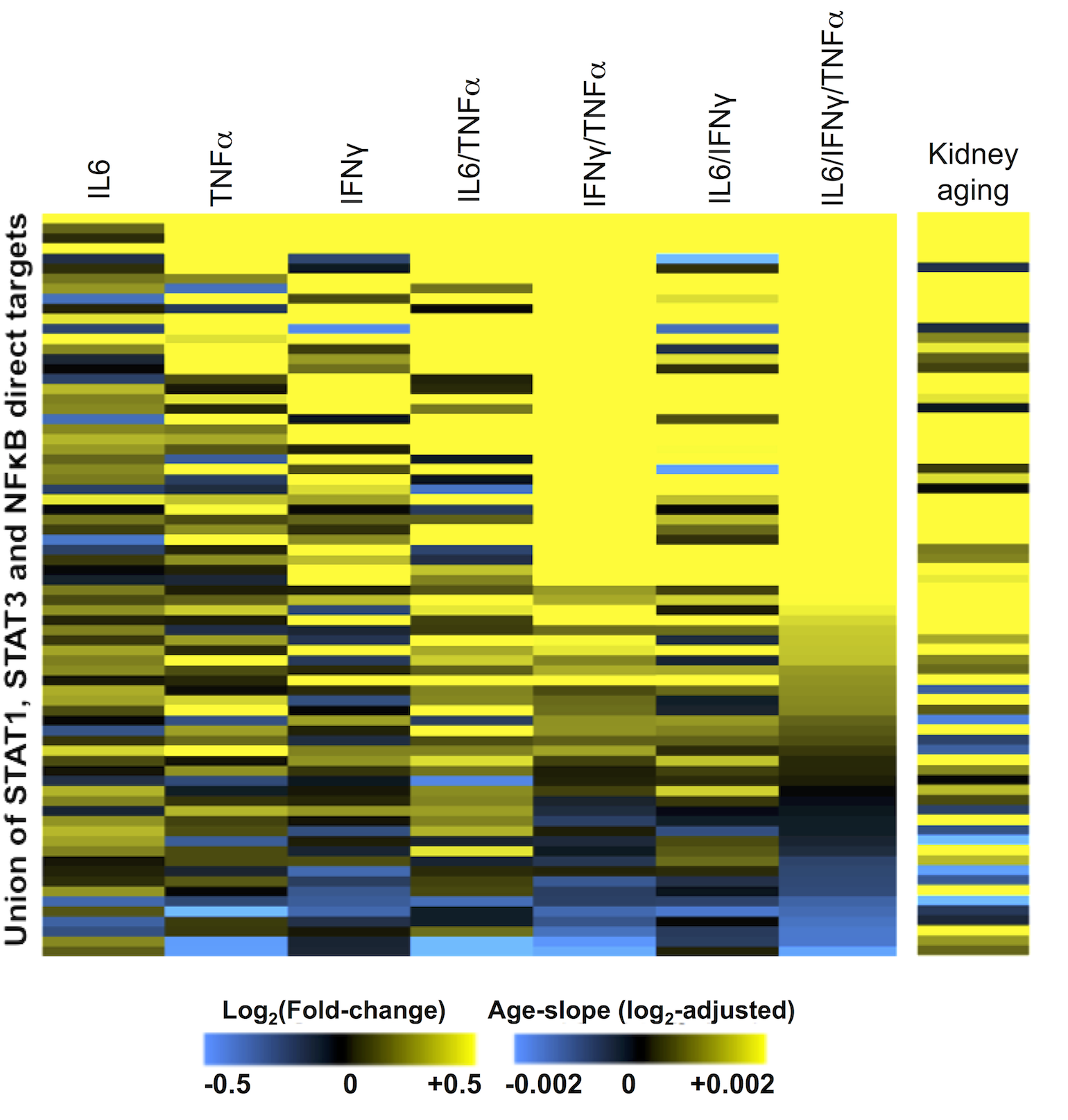

Supplement: S5 Fig — The first seven columns of the heat map shows gene expression changes in response to combinations of inflammatory cytokines. Each of the 74 rows corresponds to a gene that is a direct target of STAT1, STAT3, or NFκB. The color indicates log2 fold-changes in gene expression for the direct targets. The last column of the heat map shows the corresponding log2-adjusted beta coefficient (age-slope) for these transcripts during kidney aging, using data from Rodwell et al. 2004 [1]. (TIF) [file pgen.1005734.s005.tif]

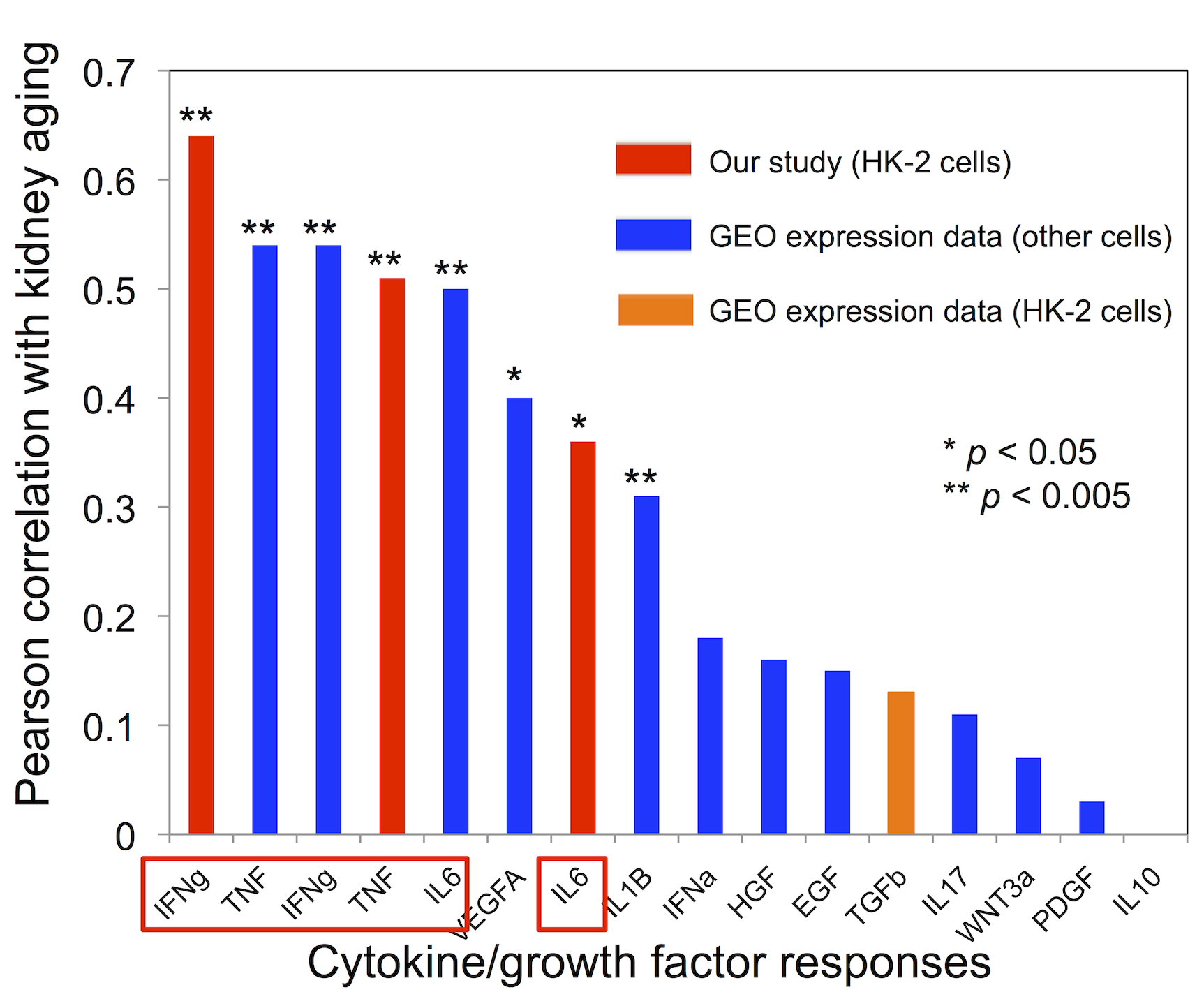

Supplement: S6 Fig — The y-axis of the histogram shows the Pearson correlations between gene expression changes following stimulation of human cells with different cytokines and growth factors indicated on the x-axis and their expression changes during kidney aging. Genes with significant differential expression at p < 0.001 were considered significant in these analyses. Three of the datasets were from the present study (red bars). The remaining microarray datasets were downloaded from GEO [20–30], and include the responses of other cells to IL-6, TNFα and IFNγ as well as the responses of diverse human cell types to 10 other cytokines or growth factors (VEGFA, Wnt3a, PDGF, IL10, TGF-ß EGF, HGF, IL17, IL1ß, IFNα). *Indicates p < 0.05, **p < 0.005 (Pearson correlation p-value). (TIF) [file pgen.1005734.s006.tif]

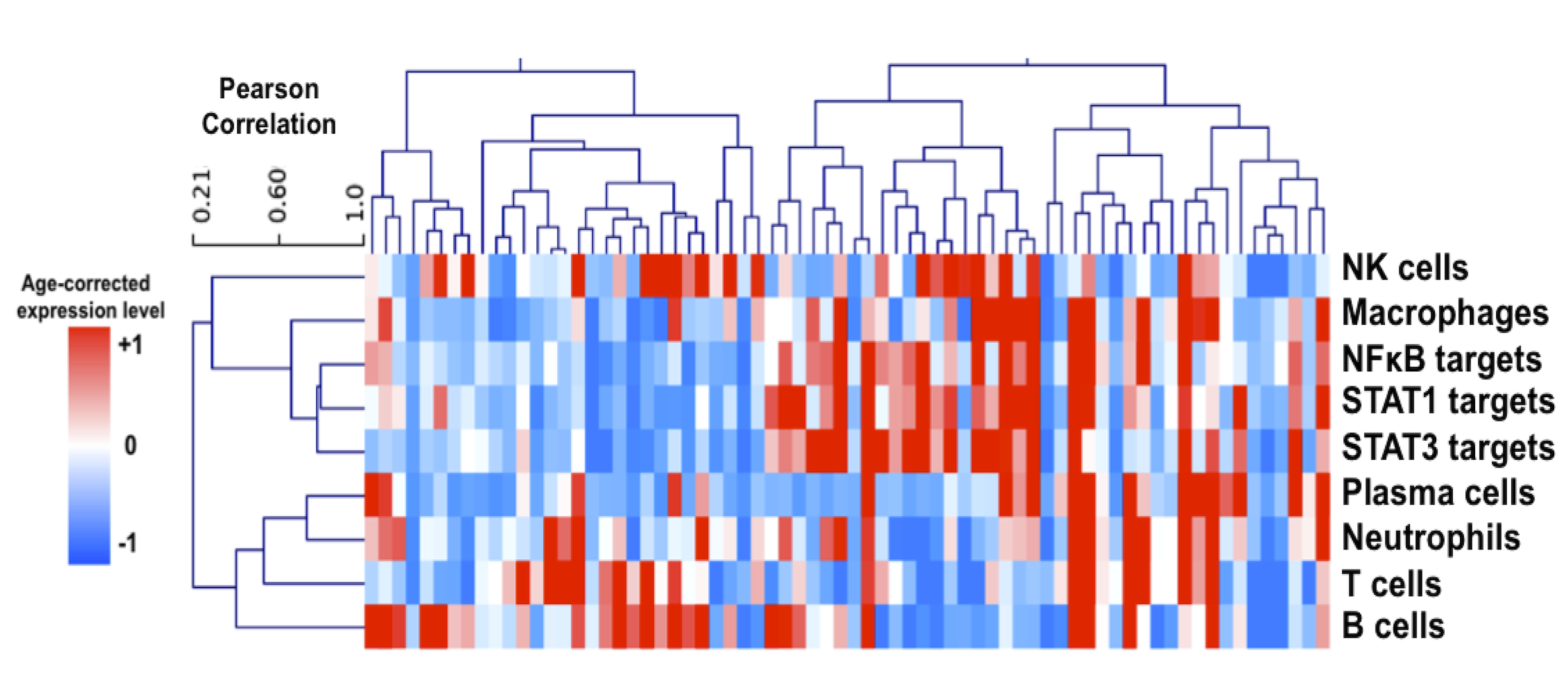

Supplement: S7 Fig — Each row in the heat map shows the estimated activity of STAT1, STAT3, NFκB (based on target gene expression) or the abundance of macrophage, B cells, T cells, NK cells, neutrophils, plasma cells (estimated by marker expression). Each column represents an individual kidney RNA sample. The color indicates the level of transcription factor activation or the abundance of cells in an individual kidney, normalized for age. The score shows whether the activity of the transcription factor or estimated abundance of immune cell types is higher or lower than the average for others at the same age. Red color indicates individuals with high relative expression or cell abundance and blue color indicates individuals with low relative expression or cell abundance compared to the age-adjusted mean. The dendrogram on the left shows that correlation of transcription factor activity is higher with estimated macrophage abundance than with the estimated abundance of other types of immune cells. (TIF) [file pgen.1005734.s007.tif]

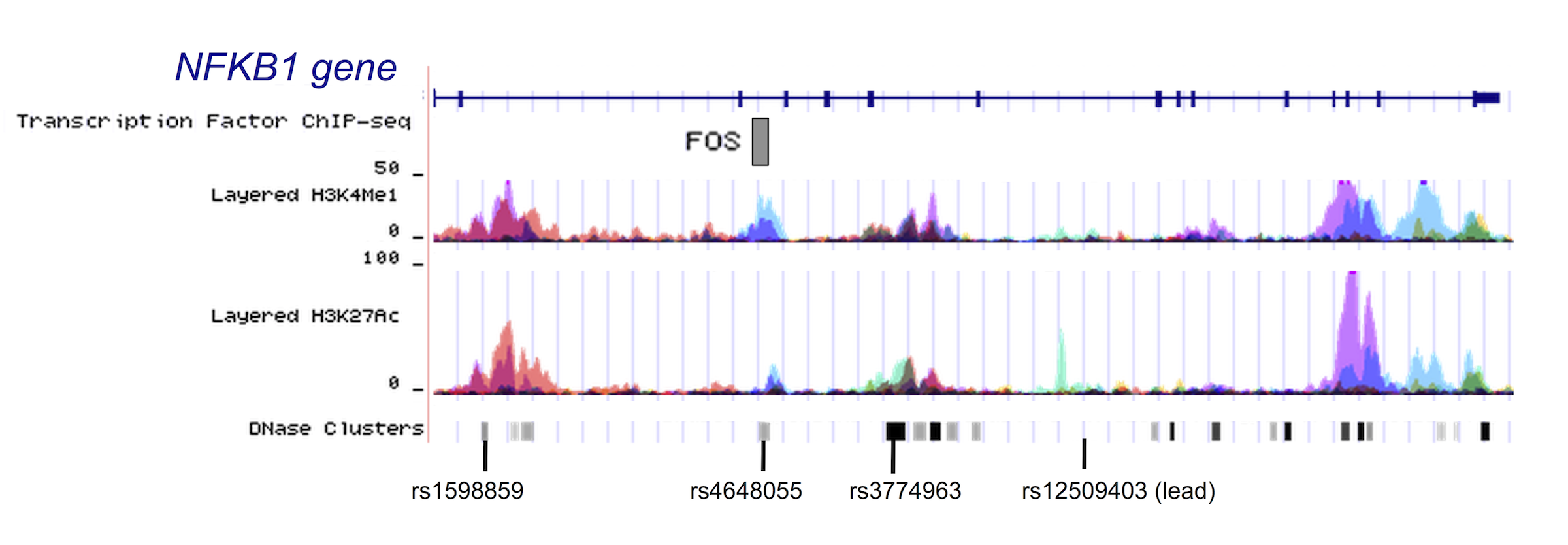

Supplement: S8 Fig — UCSC genome browser screen shot showing the NFKB1 gene (exons shown as boxes). The grey box indicates a Fos ChIP-seq binding peak that contains rs4648055. Shown below are tracks for H3K4me1 (a mark found near regulatory elements) and H3K27ac (a common mark of active enhancers). The colors of the peaks represent different cell lines. The grey and black boxes indicate Dnase I hypersensitivity clusters in the lower panel of the image. rs12509403 is the lead eQTL SNP. Shown are three SNPs linked to rs12509403 (R2 > 0.8) that may affect expression of NFKB1 as they occur within Dnase I hypersensitivity clusters, regulatory histone marks, or affect transcription factor binding. (TIF) [file pgen.1005734.s008.tif]

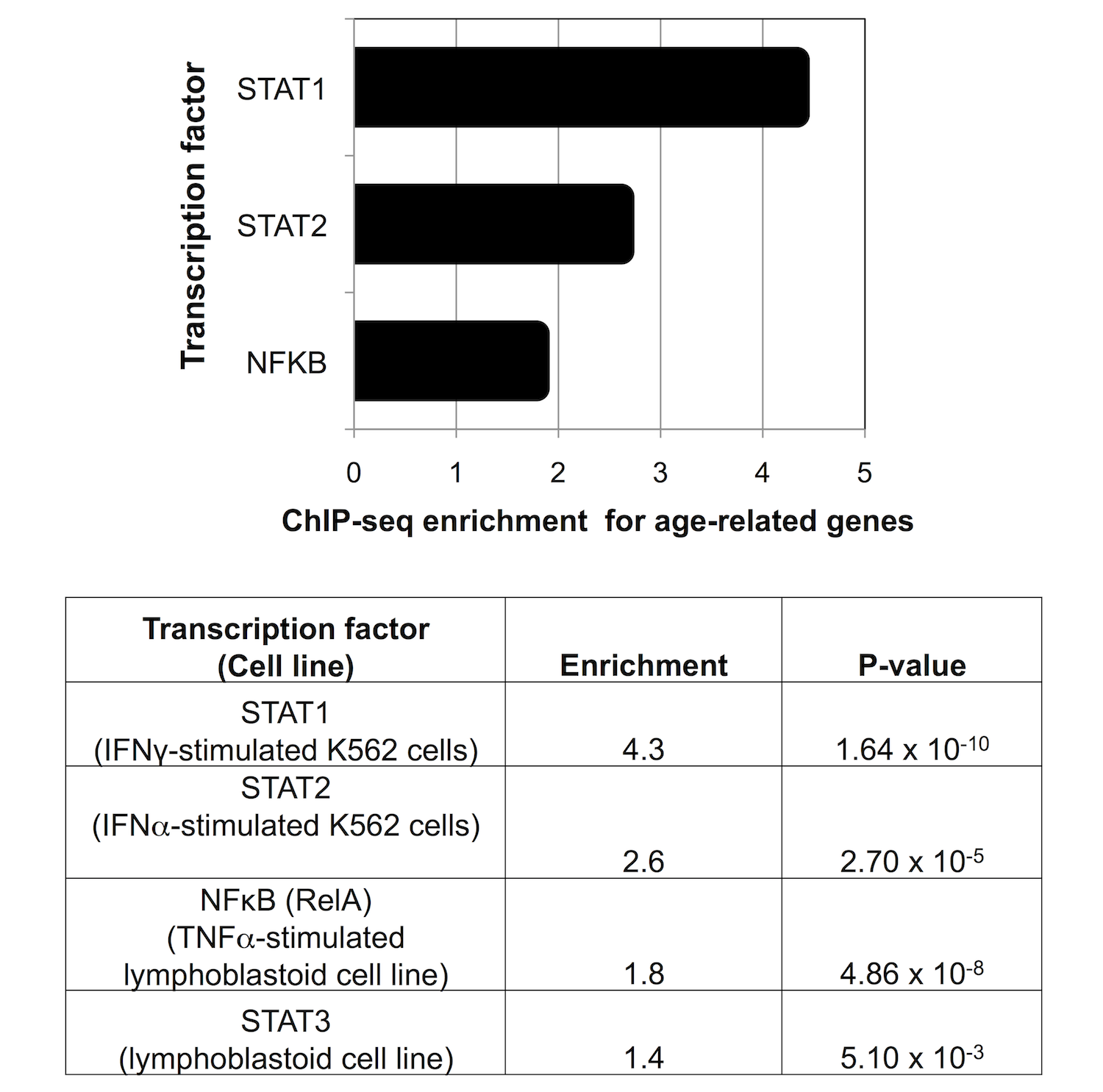

Supplement: S9 Fig — We repeated the screen of the ENCODE ChIP seq database using all transcription factor binding peaks, rather than only using binding peaks with a complexity score <81 as in Fig 1B. Top. The histogram shows the results for the three transcription factors that meet the selection criteria used in Fig 1B: enrichment p-value that is Bonferroni significant (p < 5 x 10^−5^) and a >1.5-fold enrichment. Bottom. Table shows the top four ChIP-seq datasets for enrichment for binding to kidney age-related genes along with the transcription factor and cell lines used, ranked by p-value. STAT1, STAT3 and NFκB are among the top four ChIP-seq datasets that show enrichment for binding to the kidney age-related genes. (TIF) [file pgen.1005734.s009.tif]
